# 24-h continuous non-invasive multiparameter home monitoring of vitals in patients with Rett syndrome by an innovative wearable technology: evidence of an overlooked chronic fatigue status

**DOI:** 10.3389/fneur.2024.1388506

**Published:** 2024-06-17

**Authors:** Silvia Leoncini, Lidia Boasiako, Sofia Di Lucia, Amir Beker, Valeria Scandurra, Aglaia Vignoli, Maria Paola Canevini, Giulia Prato, Lino Nobili, Antonio Gennaro Nicotera, Gabriella Di Rosa, Maria Beatrice Testa Chiarini, Renato Cutrera, Salvatore Grosso, Giacomo Lazzeri, Enrico Tongiorgi, Pasquale Morano, Matteo Botteghi, Alessandro Barducci, Claudio De Felice

**Affiliations:** ^1^Rett Syndrome Trial Center, University Hospital Azienda Ospedaliera Universitaria Senese, Siena, Italy; ^2^Neonatal Intensive Care Unit, University Hospital Azienda Ospedaliera Universitaria Senese, Siena, Italy; ^3^U.O.S.A. Programmazione e Ricerca Clinica, University Hospital Azienda Ospedaliera Universitaria Senese, Siena, Italy; ^4^Accyourate Group SpA, L’Aquila, Italy; ^5^Child Neuropsychiatry Unit, University Hospital Azienda Ospedaliera Universitaria Senese, Siena, Italy; ^6^Childhood and Adolescence Neurology and Psychiatry Unit, ASST GOM Niguarda, Milan, Italy; ^7^Epilepsy Center – Sleep Medicine Center, Childhood and Adolescence Neuropsychiatry Unit, ASST Santi Paolo e Carlo, San Paolo Hospital, Milan, Italy; ^8^Child Neuropsychiatry Unit, IRCCS Istituto Giannina Gaslini, Genova, Italy; ^9^Department of Neurosciences, Rehabilitation, Ophthalmology, Genetics and Maternal and Child Health (DINOGMI), University of Genova, Genova, Italy; ^10^Child Neuropsychiatry Unit, University Hospital “G. Martino”, Messina, Italy; ^11^Department of Biomedical and Dental Sciences and of Morphological and Functional Imaging (BIOMORF), University of Messina, Messina, Italy; ^12^Pneumology and Cystic Fibrosis Unit, Academic Department of Pediatrics, Bambino Gesù Children’s Hospital, Rome, Italy; ^13^Department of Molecular and Developmental Medicine, University of Siena, Siena, Italy; ^14^Pediatrics Unit, University Hospital Azienda Ospedaliera Universitaria Senese, Siena, Italy; ^15^Department of Life Sciences, University of Trieste, Trieste, Italy; ^16^Croce Rossa Italiana, Rome, Italy; ^17^Department of Clinical and Molecular Sciences – Experimental Pathology Research Group, Università Politecnica delle Marche, Ancona, Italy; ^18^Medical Physics Activities Coordination Centre – Alma Mater Studiorum – University of Bologna, Bologna, Italy; ^19^Italian Color Solutions I.C.S. S.r.l., Pistoia, Italy

**Keywords:** Rett syndrome, cardiorespiratory monitoring, wearable devices, sleep–wake cycle, exercise fatigue

## Abstract

**Background:**

Sleep is disturbed in Rett syndrome (RTT), a rare and progressive neurodevelopmental disorder primarily affecting female patients (prevalence 7.1/100,000 female patients) linked to pathogenic variations in the X-linked methyl-CpG-binding protein 2 (*MECP2*) gene. Autonomic nervous system dysfunction with a predominance of the sympathetic nervous system (SNS) over the parasympathetic nervous system (PSNS) is reported in RTT, along with exercise fatigue and increased sudden death risk. The aim of the present study was to test the feasibility of a continuous 24 h non-invasive home monitoring of the biological vitals (biovitals) by an innovative wearable sensor device in pediatric and adolescent/adult RTT patients.

**Methods:**

A total of 10 female patients (mean age 18.3 ± 9.4 years, range 4.7–35.5 years) with typical RTT and *MECP2* pathogenic variations were enrolled. Clinical severity was assessed by validated scales. Heart rate (HR), respiratory rate (RR), and skin temperature (SkT) were monitored by the YouCare Wearable Medical Device (Accyourate Group SpA, L’Aquila, Italy). The average percentage of maximum HR (HRmax%) was calculated. Heart rate variability (HRV) was expressed by consolidated time-domain and frequency-domain parameters. The HR/LF (low frequency) ratio, indicating SNS activation under dynamic exercise, was calculated. Simultaneous continuous measurement of indoor air quality variables was performed and the patients’ contributions to the surrounding water vapor partial pressure [P_H2O_ (pt)] and carbon dioxide [P_CO2_ (pt)] were indirectly estimated.

**Results:**

Of the 6,559.79 h of biovital recordings, 5051.03 h (77%) were valid for data interpretation. Sleep and wake hours were 9.0 ± 1.1 h and 14.9 ± 1.1 h, respectively. HRmax % [median: 71.86% (interquartile range 61.03–82%)] and HR/LF [median: 3.75 (interquartile range 3.19–5.05)] were elevated, independent from the wake–sleep cycle. The majority of HRV time- and frequency-domain parameters were significantly higher in the pediatric patients (*p* ≤ 0.031). The HRV HR/LF ratio was associated with phenotype severity, disease progression, clinical sleep disorder, subclinical hypoxia, and electroencephalographic observations of multifocal epileptic activity and general background slowing.

**Conclusion:**

Our findings indicate the feasibility of a continuous 24-h non-invasive home monitoring of biovital parameters in RTT. Moreover, for the first time, HRmax% and the HR/LF ratio were identified as potential objective markers of fatigue, illness severity, and disease progression.

## 1 Introduction

Rett syndrome (RTT) (OMIM #312750) is a rare X-linked neurodevelopmental disorder that mainly affects female individuals (prevalence 7.1/100,000 female patients) (1, 2). RTT is a multisystemic disease (3) caused (in over 90–95% of cases) by a *de novo* loss of function mutations in the methyl-CpG-binding protein 2 gene (*MECP2*) (4). The classical clinical presentation of the disease (1) is characterized by a period of 6 to 18 months of apparently normal neurodevelopment, followed by an early neurological regression, with a progressive loss of acquired cognitive, social, and motor skills in a typical 4-stage neurological regression pattern (5, 6).

RTT is a complex disease with a multifaceted clinical appearance (3). Main neurological concerns are epilepsy (7), transient autistic phase (8), gait ataxia (9), intellectual deficit (9), autonomic nervous system dysfunction (ANSD) (3), sleep problems (3, 10–12), abnormal movements, and behavior disturbances (3). Other common comorbidities are represented by breathing dysfunction (3, 13–17), gastrointestinal (GI) problems (3), scoliosis (3, 18), and bone deficiency (osteopenia/osteoporosis with high risk of fractures) (3).

Autonomic nervous system dysfunction (ANSD) is a relevant contributor to some of the clinical features in RTT patients that leads to multiple impairments ranging from anxiety, GI dysfunction, breathing disturbances, peripheral vascular control alteration (19) to sleep issues (3), heart rate variability (HRV) (20–22), and increased risk of sudden death (23, 24).

Sleep pattern is severely disturbed in RTT. The prevalence of sleep disorders in these patients is reported to be approximately 80–94% (3, 10–12). Clinical manifestations such as night-time laughter, bruxism, diurnal napping, night screaming, nocturnal seizures, night terrors (3, 10), nocturnal waking (3), daytime sleepiness (12), and disorders of initiating and maintaining sleep (3) are frequent and linked to the patients’ age and mutation type (3, 10).

ANSD can result in cardiac abnormalities and reduced HRV in RTT patients, and varies with bradycardia (25), subclinical myocarditis (26), ventricular tachyarrhythmia (27), prolonged QTc interval (28), cardiac repolarization parameters (28, 29), cardiorespiratory coupling measurements (21, 23, 30–32), and reduced HRV (20–23). Dysautonomia and breathing dysrhythmia may interfere with physical activity and cause poor weight gain, impacting fatigue (3). A low level of tissue aerobic respiration has been reported in RTT patients exposed to exercise, thus indicating that RTT patients could get tired quicker with a lower-than-normal physical stamina compared to the control group (33). Interestingly, exercise fatigue is a hallmark feature in the experimental models recapitulating RTT (34, 35) believed to be related to peripheral MeCP2 deficiency (36) and mitochondrial dysfunction (37). Development of medical technology has allowed the use of wearable devices also in the healthcare field (38, 39). HRV is used to examine the autonomic cardiac function under physiological and pathological conditions (40, 41) and estimate fatigue (42, 43).

To date, a few studies have been carried out in RTT patients regarding the feasibility of monitoring medical vital signs in a home environment by non-invasive wearable devices during daytime and nighttime (19, 44–48). Previously, vital signs and behavioral features have been mainly evaluated by questionnaires administered to caregivers (10, 11) or by invasive methods, such as polysomnography (PSG) (49, 50) during hospitalization.

Moreover, to the best of our knowledge, no clinical studies are found to exist evaluating longitudinal multiparameter by consistently monitoring an extensive time and/or indirectly targeting the assessment of fatigue in RTT patients. Recently, an innovative wearable technology with polymeric non-invasive sensors printed into a textile fabric has been developed, which allows a real-time monitoring of biovital and kinetic parameters with high ergonomic quality (51, 52).

This new technology, with continuous monitoring of the health status in everyday context, represents an improvement of the individual psychophysical wellbeing and health rescue purposes (51, 52). The aim of the present study was to test the feasibility of a continuous non-invasive 24-h home monitoring of biovital parameters in RTT patients using a non-invasive, innovative wearable technology.

## 2 Materials and methods

### 2.1 Participants

The clinical trial was set as a pilot monocentric study. The study was approved by the Ethical Committee of Siena University Hospital, Azienda Ospedaliera Universitaria Senese (AOUS), Siena, Italy (Approval code n. 23573, date 16 January 2023).

To meet the established objectives, the study was designed to recruit different RTT patients as a function of age, i.e., pediatric (aged 3–13 years) vs. adolescent/adult (aged >13 years). Inclusion criteria were as follows: i) female positive for pathogenic *MECP2* gene mutation (4) and clinical criteria for typical RTT (6), ii) age ≥ 3 years and body weight ≥ 10 kg (according to the indications for the approved use of the YouCare medical device in Italy); and iii) signed written informed consent from the patients’ parents/caregivers/legal guardians. Similarly, exclusion criteria were as follows: i) patients without RTT clinical criteria according to the guidelines; ii) patients negative for pathogenic *MECP2* gene mutation or mutation in non-*MECP2* genes (i.e., *CDKL5* and *FOXG1*); iii) male RTT; and iv) positive history of contact dermatitis and/or known hypersensitivity against the textile or other materials of the sensorized T-shirts. Parents/caregivers/legal guardians of patients meeting inclusion/exclusion criteria were requested to participate during a regular follow-up at the AOUS RTT center and a written informed consent was obtained.

A total of 10 RTT patients were enrolled (mean age 18.3 ± 9.4 years, range 4.7–35.5 years) with a clinical diagnosis of typical RTT and proven *MECP2* gene mutation. All participants received a structured evaluation by an expert to assess clinical severity and anthropometric measurements as well as specific body measures (i.e., chest and abdomen) to guarantee the correct fitting of *t*-shirts worn by the patients. The corresponding z-scores for head circumference and body mass index were calculated based on validated RTT-specific growth charts (53). To consider the possible effect of the presence of scoliosis and epilepsy on respiratory dysfunction, data regarding the coronal Cobb angle, presence of epilepsy, seizure frequency, and anti-seizure medication (ASM) therapy were recorded. Scoliosis severity was rated: mild (Cobb angle 10°–20°), moderate (Cobb angle <40°), or severe (≥ 40°), according to Killian et al. (18). Clinical stages distribution was as follows: stage II (*n* = 2), stage III (*n* = 3), and stage IV (*n* = 5). The study took place at the Rett Syndrome National Reference Center of the AOUS.

The duration of the study was 16 weeks, which included one monthly visit to the center. Sensitive clinical data were anonymized by assigning a randomly generated integer code to each RTT patient. An electroencephalographic (EEG) examination was performed before the baseline visit according to the standard clinical follow-up of the patients. The systems used were a System Plus Evolution [Micromed SpA, Mogliano Veneto (TV) Italy], with 32 channels for pediatric patients (recording with gain 100 μv/cm, high pass filter 1.6 Hz; low pass filter 50 Hz) and Mizar 40 with 40 channels for adult patients (2 kHz recording and storage at 128 Hz, filter 0.3 s-70 Hz). The following were recorded in the study: waking, falling asleep, sleeping, awakening, and waking with light stimulation tests. In particular, EEG multifocal epileptiform activity and severe general background slowing were considered key EEG alterations, given their high frequency and relevance in the clinical course of the disease. At the inclusion visit, continuous and non-invasive measurements of oxyhemoglobin saturation (SpO_2_) were evaluated using pulse oximetry motion artifact-free technology Masimo Radical 7 (Masimo SET; Masimo Corp., Irvine, CA, United States).

### 2.2 Clinical severity

The clinical severity of the illness was assessed by validated scales for RTT, which were Clinical Severity Score (CSS) (54), Motor Behavioral Assessment Scale (MBAS) (55), Rett Syndrome Behavior Questionnaire (RSBQ) (56), and Multi-System Profile of Symptoms Scale (MPSS) (57). CSS is a specific Likert scale of 13 items designed to assess the natural history of key symptoms (i.e., age of onset of regression, somatic growth, head growth, independent sitting, ambulation, hand use, scoliosis, language, non-verbal communication, respiratory dysfunction, autonomic symptoms, onset of stereotypies, and seizures). The MBAS is designed to survey movement abnormalities, in particular extrapyramidal symptoms, behavioral problems, and abnormal physiological features in individuals with RTT. It is a Likert checklist of 37 items subdivided into social and communication skills and adaptive behaviors (MBAS I), orofacial and respiratory abilities (MBAS II), and motor abilities/physical signs (MBAS III). The RSBQ, a Likert checklist of 45 items, measures behavioral and emotional features and movement abnormalities. RSBQ consists of 8 subdomains: general mood, breathing, hand behavior, repetitive face movements, body rocking and expressionless face, night-time behavior, fear/anxiety, and walking/standing. The MPSS is a Likert scale utilized to evaluate the frequency of symptoms in RTT patients. The scale is divided into 12 main areas, including mental health, cardiac dysfunction, autonomic dysfunction, communication problems, social behavior problems, emotional engagement, gastrointestinal problems, motor skills, neurological problems, orofacial problems, breathing, and sleep disorder. To evaluate possible changes in some features of RTT, the scale is integrated with 5 supplementary areas such as immunity dysfunction, infection, sensory problems, endocrine problems, skeletal problems, and dermatological problems.

### 2.3 Clinical sleep evaluation

To evaluate the effect of the sleep–wake cycle, the caregivers were asked to complete a patient diary in which the 24 h were categorized as wake-up, wakefulness, napping, night sleep onset, and night bedtime. Sleep quality was indirectly evaluated by the Sleep Disturbance Scale for Children Questionnaire (SDSC) (58). The questionnaire consists of 26 items grouped into 6 subscales related to the major sleep complaints in pediatric age: disorders in initiating and maintaining sleep (DIMS), sleep breathing disorders (SBD), disorders of arousals/nightmares (DA), sleep/wake transition disorders (SWTD), disorders of excessive somnolence (DOES), and sleep hyperhidrosis (SHY). Total sleep time and sleep onset latency were classified according to Bruni et al. (58). Co-sleeping behavior frequency of patients was also explored.

### 2.4 Quality of life

The Quality-of-Life Inventory-Disability (QI-Disability) is a 32-item questionnaire assessing the quality of life of children with intellectual disability (59, 60). The questionnaire is comprised of six domains: social interaction (7 items), positive emotions (4 items), negative emotions (7 items), physical health (4 items), leisure and the outdoors (5 items), and independence (5 items). The daily events diary is a Likert scale that evaluates the intensity of 6 items (hands stereotypes, seizures, apparent suffering aspect, irritability/agitation, energy, and attention). The diary is completed by the parents/legal tutors daily.

### 2.5 Continuous multiparameter monitoring by YouCare medical wearable device

Accyourate wearable technology consists of textile garments equipped with innovative non-invasive polymeric sensors on the clothing via ink-jet printing. The wearability and ergonomic characteristics of these devices allow a new way of detecting biovital and kinetic parameters by providing dynamic measurements of individual status in real-time. The YouCare “Smart T-shirt” is made of organic and washable fabric equipped with printed sensors embedded in the garment. YouCare is a certified medical device [YouCare, EC marking for medical device (MD type IIa), Accyourate Group SpA, L’Aquila, Italy] having the appearance of a T-shirt that covers the upper part of the chest. It was used to monitor several biovital parameters for 24 h in a home setting and under ordinary living conditions and activities. Measured and calculated parameters included heart rate (HR), respiratory rate (RR), skin temperature (SkT) (51, 52), and heart rate variability (HRV, expressed as time-domain and frequency-domain parameters) (61). Specifically, the epoch size for the HRV analysis [Root mean square of successive RR interval differences (RMSSD)] was 10 s, which is the accepted minimum gold standard (61).

In addition, the kinetic parameters detected by the sensors provide dynamic measurements of individual status in real-time, given the comfortable wearability and favorable ergonomic features of the YouCare device. The signals taken from the sensors are tandemly digitized and processed by a miniaturized wearable control unit, which records the data and sends them to a smartphone equipped with the YouCare app [Accyourate Group SpA, L’Aquila, Italy] via Bluetooth. This process, in turn, carries out real-time data received and exchanged with the Accyourate cloud platform, which centralizes and stores the post-processing of the data collected. The cloud platform analyses the data through dedicated, proprietary software and algorithms. The raw data are analyzed on the cloud with the exception of a few plots analyzed on the device (i.e., smartphone) for the conversion of the skin temperature data. To identify artifacts, the algorithm reported (52) was used and subsequently discarded from the analysis.

YouCare has been proven effective in several clinical trials: i) the sports sector (both competitive and non-competitive); ii) protection of health in advanced age cohorts; iii) monitoring of fragile patients after COVID-19 infection; iv) collection of health datasets based on ECG measurements and their clinical interpretation; v) validation of innovative diagnostic methodologies in comparison with traditional ones; vi) applications to the military and industrial fields; and vii) protection of operators in high-risk working environments (62). Sinus rhythm was not discriminated from the non-sinus rhythm in the current analyses, where an interbeat distance of 200 ms to 1800 ms was considered acceptable for analysis.

The derived HRV time-domain parameters included RMSSD, Standard Deviation (SD) of all NN intervals (i.e., interbeat intervals from which artifacts have been removed) (SDNN), SD of R-R intervals (SDRR), RMSSD to MeanNNI ratio (CVSD), percentage of successive R-R intervals differing by more than 20 ms (pNNI-20), percentage of successive R-R intervals differing by more than 50 ms (pNNI-50), SD of successive differences between NN (SDSD), Mean of NN (M-NNI), and SDNN divided by mean NN (CVNNI). In addition, the derived HRV frequency-domain parameters were as follows: Low-Frequency power (LF), High Frequency power (HF), LF/HF ratio, normalized LF power (LFnu), normalized High-Frequency power (HFnu); and total spectral power.

The measurements of breast circumference, solar (celiac) plexus circumference, and shoulder-height solar plexus were also obtained in the study to design the individual clothing. The Accyourate clothing designer assigned the corresponding sensorized textiles with the specific fitting. At the baseline visit, an experimental kit was provided to each RTT patient’s family, consisting of the following items: two YouCare T-shirts; a control unit with an anonymous code serial number; and a series of accessories, including a battery charger, a USB cable type C, and the use of the YouCare APP. Each medical device was paired to the control unit with an individual patient code number known to the clinical staff only. To obtain reliable biovital results (i.e., closely reflecting the real daily life conditions of the patients) coupled with the need to test the compliance of the patients–caregiver pairs, the biovital monitoring plan was carefully scheduled with the families. In particular, the continuous multiparameter monitoring schedule was either 4 sessions weekly (week 1, week 3) or 3 (week 2, week 4) sessions of 12 h duration each. During the weekend days from week 1 to 4, at least one session of 24 h was performed. The recording sessions, with a cadence of 3 sessions weekly, were therefore extended to 24 h from week 5 to week 12 (i.e., end of the study). To evaluate the effect of daily activities on biovital monitoring, the caregivers were also asked to complete a patient diary.

### 2.6 Measurement of exercise fatigue: percentage of maximum heart rate (Max HR %) and heart rate to HRV Low-Frequency power (HR/LF ratio)

From the continued monitoring of HR records, the percentage of maximum heart rate (HRmax %) was calculated as an indirect proxy index of fatigue and categorized according to the target heart rate. According to the American Heart Association definition (63), HR% in RTT girls was categorized based on maximum heart rate (Max HR), low exercise intensity (Max HR% <50), moderate exercise intensity (Max HR%: 50% to approximately 70%), vigorous exercise intensity (Max HR%: 70% to approximately 85%), and maximal exercise intensity (Max HR% > 85%). Given the consistently elevated Max HR values observed in our study, we reasoned that RTT patients are usually considered fragile and motor-impaired paradoxically compared to young athletes under active dynamic exercise. The accepted model of autonomic control of HR during dynamic exercise indicates that the initial increase is entirely attributable to the withdrawal of parasympathetic nervous system (PSNS) activity and subsequent increase in HR is entirely attributed to increased cardiac sympathetic activity.

Results here indicate that: (i) an increase in exercise workload-related HR is not caused by a total withdrawal of the PSNS, which follows an increase in sympathetic tone; (ii) reciprocal antagonism is key to transition from vagal to sympathetic dominance, and (iii) resetting of the arterial baroreflex (64) causes an immediate exercise-onset reflexive increase in HR, which are parasympathetically mediated and follows a slower increase in sympathetic tone as workload increases (65). It has been previously reported that the LF/HF ratio does not accurately measure cardiac sympathovagal balance (66). On the other hand, the HR/LF ratio appears to reflect sympathetic nervous activity and metabolic responses during incremental cycling exercise in healthy adults (67). Indeed, HR during exercise is determined by the interplay between sympathetic and parasympathetic nervous activity. As exercise intensity increases, the dominance of autonomic nervous activity is shifted from parasympathetic to sympathetic nervous activity, and HR linearly increases, which is considered to be caused by baroreflex resetting (64, 65). The baroreflex efferent is mediated through both sympathetic and parasympathetic nervous signals that can influence the LF component of HRV (68). Moreover, the LF component is reported to be more strongly influenced by parasympathetic activity than sympathetic activity (69). Hence, it has been hypothesized that a change in HR corrected by the LF components could reflect sympathetic nervous activation by showing the relative decrease in the ability of the parasympathetic nervous system to modulate HR by baroreflex resetting during exercise. For these reasons, we decided to test the interpretive potential of the HR/LF ratio in the continuous home monitoring in our RTT patients.

### 2.7 Indoor Air Quality (IAQ) parameters and environmental data

An NHC01 weather station with indoor sensors (Netatmo, Boulogne-Billancourt, France) was also provided at the baseline visit. Key environmental data included relative humidity (range: 0–100), air temperature (range: 0–50°C), air carbon dioxide (CO_2_)(range: 0–5,000 ppm), and noise (range: 35–120 dB). The system was placed in the patients’ living spaces during the day and in the patients’ bedrooms during sleeping time (no co-sleeping together with parents and/or siblings was reported for this study). For the environmental recordings, only the patients’ hours in-home were considered. For the indoor air quality parameters, home monitoring time covered the 24 h, excluding the hours spent in schooling/day-time centers, rehabilitation (physiotherapy, hydro-kinesitherapy, speech therapy, music therapy, hippotherapy, and other rehabilitation therapies), and outdoor daily activities. The rationale behind evaluating these environmental data was to investigate their possible relationship with biophysical parameters. For example, important biophysical parameters, such as the RR and HR, can have a significant influence on the gas volume exhaled by the patient. Thus, from a theoretical standpoint, it is plausible that the CO_2_ and H_2_O molar concentrations measured in the patient’s room may be in relation to several biophysical parameters.

### 2.8 Indirect estimation of the total water saturation vapor pressure

Saturation vapor pressure is useful for converting the relative humidity monitored in the room to the partial pressure of H_2_O vapor $P_{H2O}^{0}\left(T\right)$ . For this, the August–Roche–Magnus formula (70) was applied as shown below:


(1) $$P_{H2O}^{0}\left(T\right)≅6.1094e^{\frac{17.625T}{243.04+T}}$$

The above equation contains the Celsius temperature T , and estimates of the water vapor partial pressure in hPa or mbar. Environmental temperatures in the sleeping room have been recorded for each patient, allowing the water saturation vapor pressure to be estimated.

### 2.9 Indirect estimation of the patient contribution to the water vapor pressure

The measured humidity is given by the sum of two contributions: the environmental humidity, $R_{H}\left(env\right)$ , and the contribution originated by the patient, $R_{H}\left(pt\right)$ . Both parameters can change with time. Nonetheless, we assumed a value of the environmental contribution to be constant. With this assumption, we can write:


(2) $$R_{H}=R_{H}\left(env\right)+R_{H}\left(pt\right)$$

(3) $$R_{H}\left(env\right)<\min{\left\{R_{H}\right\}}_{t}$$

We corrected the recorded relative humidity by subtracting the 80% of the minimum $R_{H}$ detected in the period of patient observation, which is a fraction of the measured $R_{H}$ that would be ascribed to the unperturbed environment. It is presumed that a large fraction of the recorded minimal humidity is due to the environment. This heuristic and arbitrary correction is outlined in the following relationship:


(4) $$R_{H}\left(pt\right)\sim R_{H}-0.8\cdot \min{\left\{R_{H}\right\}}_{t}$$

(5) $$R_{H}\left(env\right)\sim 0.8\cdot \min{\left\{R_{H}\right\}}_{t}$$

Using the water saturation vapor pressure estimated with Eq. 1 and the relative humidity $R_{H}\left(pt\right)$ of Eq. 4, it was possible to retrieve the water vapor partial pressure $P_{H2O}$ due to the patient presence in the sleeping room, as shown by the next equation:


(6) $$P_{H2O}\left(pt\right)\approx \frac{P_{H2O}^{0}\left(T\right)\cdot R_{H}\left(pt\right)}{100}=\frac{P_{H2O}^{0}\left(T\right)\cdot \left[R_{H}-0.8\cdot \min{\left\{R_{H}\right\}}_{t}\right]}{100}$$

The conversion of relative humidity to partial pressure is discussed in the following reports (71, 72).

### 2.10 Indirect estimation of the patient contribution to the CO_2_ abundance and partial pressure

Carbon dioxide is a fixed mixing ratio component of the Earth’s atmosphere, which is almost invariant with height and geographical location. Variations with geographic position do not exceed ±5% of its global average as reported by a CO_2_ global mapping research (73). To date, an average CO_2_ concentration close to 420 parts per million (ppm) is to be expected (74).

The recorded CO_2_ concentration $X_{CO2}^{0}$ was therefore corrected for this expected value, finding the CO_2_ contribution $X_{CO2}\left(pt\right)$ originated by the patient:


(7) $$X_{CO2}\left(pt\right)≅X_{CO2}^{0}-420$$

It can easily be shown that the CO_2_ partial pressure $P_{CO2}$ can be obtained directly from the total atmospheric pressure $P_{air}\left(tot\right)$ , another parameter measured by our environmental sensors, multiplied by its abundance $X_{CO2}$ . Similarly, considering the patient’s contribution $X_{CO2}\left(pt\right)$ to the total CO_2_ abundance, the patient contribution to the CO_2_ partial pressure is retrieved. The equation below shows this property:


(8) $$P_{CO2}\left(pt\right)=X_{CO2}\left(pt\right)\cdot P_{air}\left(tot\right)$$

### 2.11 Estimation of molarity (molar concentration) of H_2_O and CO_2_

Molarity $\rho_{n}$ estimation for a given molecule can be obtained from its partial pressure using the ideal gas law, which states:


(9) $$P\cdot V≅nRT↔\frac{P}{RT}=\frac{n}{V}$$

(10) $$\rho =\frac{n}{V}=\frac{P}{RT}$$

In the equations above, V is the gas volume, P its pressure, n the mol number, and R the ideal gas constant. Data collected by the smart t-shirt and the available environmental sensors allow us to estimate the molar concentration ρ . Moreover, it is possible to estimate the mol number n in the sleeping room by multiplying ρ by V:


(11) $$N=\frac{PV}{RT}$$

The estimates of ρ and N are easily obtained for both the water vapor and the CO_2_, obtaining $\rho_{H2O}$ , $\rho_{CO2}$ , $N_{H2O}$ , and $N_{CO2}$ respectively. It is noted that for all these four parameters, we have preferred to estimate the patient’s contribution relative to her body mass (e.g., $\rho_{H2O}/M$ ), assuming these contributions to be proportional to the patient’s mass. This normalization makes it easier to investigate possible relationships between the above parameters with intensive biophysical data such as HR and BR. Combining Eqs 6, 10, we can find the final expression for the patient contribution $\rho_{H2O}$ to the water vapor molarity as stated per unit of body mass:


(12) $$\rho_{H2O}=\frac{P_{H2O}^{0}\left(T\right)\cdot \left[R_{H}-0.8\cdot \min{\left\{R_{H}\right\}}_{t}\right]}{100\cdot RT}$$

Including in Eq. 12 the August–Roche–Magnus formula, we obtain:


(13) $$\rho_{H2O}=\frac{6.1094e^{\frac{17.625T}{243.04+T}}\cdot \left[R_{H}-0.8\cdot \min{\left\{R_{H}\right\}}_{t}\right]}{100\cdot RT}$$

Similarly, Eqs 7, 8, 10 provide the relationship shown in the equation below:


(14) $$\rho_{CO2}=\frac{P_{air}\left(tot\right)\cdot \left[X_{CO2}^{0}-420\right]}{RT}$$

### 2.12 Estimation of heat and heat flux

An important parameter to be considered is the heat flux $\dot{Q}$ from the body of the patient to the environment, which has a lower temperature than the patient’s skin. This (heat) energy loss has to be compensated by the patient body, thus bringing possible correlations among $\dot{Q}$ and various biophysical parameters such as HR, BR, and so forth. The heat flux $\dot{Q}$ is driven by two main body parameters: the temperature difference ( ΔT ) between the skin and the environment and the total body area A , as shown in the equation below.


(15) $$\dot{Q}=k\cdot A\cdot \Delta T$$

where k is the thermal conductivity at the body–air interface. We estimated ΔT by utilizing the temperature data measured by the environmental and wearable sensors and A as the body surface area (BSA) by adopting Mosteller’s equation (75) that is shown in the following Eq. 16.


(16) $$A≅BSA=\sqrt{\frac{H\cdot M}{36}}$$

In this relationship, H is the patient’s height and M the patient’s mass.

### 2.13 Indoor Air Quality (IAQ) data analysis

As previously reported, the sensors collecting the IAQ data (RH, air pressure, CO_2_ concentration, temperatures, and so forth) were located in different places within the patient’s room. This circumstance is relevant when the diffusivity of air constituents is taken into consideration. The various chemical species exhaled by the patient reach the sensors with a different time delay, a circumstance that may affect the time-resolved correlation among the parameters discussed so far. As an example, the CO_2_ is preferentially accumulated in the lower strata of the room atmosphere; hence, its measurement would depend on the exact positioning of the related sensor with respect to the patient position. A possible difference in height between the patient and the CO_2_ sensor would heavily influence the related readings. Therefore, we have considered the time-averaged value of any parameters where possible instead of investigating their relationships with time-resolved data.

Time averages were computed starting from time-resolved data estimated by Eqs 1–16. Several extensive data such as the mean of patient contribution to the water vapor concentration ( ${\bar{\rho }}_{H2O}$ ) have been normalized to their mass to make easier comparisons with other intensive biophysical parameters (e.g., HR and RR and their time averages $\bar{HR}$ and $\bar{RR}$ ).

### 2.14 Statistical data analysis

All variables were tested for normal distribution (D’Agostino-Pearson test), and data are presented as means ± standard deviation (M ± SD) or medians and interquartile range for continuous normal distribution and non-Gaussian variables, respectively. Mann–Whitney rank sum test (continuous non-normally distributed data), chi-square statistics (categorical variables with a minimum number of cases per cell ≥5), or Fisher’s exact test (categorical variables with a minimum number of cases per cell of 0.5) was accepted to indicate good discrimination. Relationships between variables at univariate analysis were tested using linear regression analysis or Spearman’s rank correlation. Analysis of variance was performed by one-way ANOVA or Kruskal–Wallis test, as required. To identify the discriminative power of potential predictor variables on dependent variables of interest, a receiver operating characteristic (ROC) curve analysis was performed. To test independent predictor variables for a dependent variable, stepwise multiple regression analysis models (significance entry criterion *p* < 0.05, with removal criterion *p* > 0.1) and stepwise multivariate logistic regression were tested considering the normal distribution of residuals by Kolmogorv–Smirnov test. The MedCalc version 20.013 statistical software package (MedCalc Software Ltd., Ostend, Belgium; https://www.medcalc.org 2021) was used for data analysis, and a two-tailed *p* < 0.05 was considered significant.

## 3 Results

### 3.1 Demographics, biometrics, and clinical features

Demographics, biometrics, and clinical features of the examined RTT patients (*n* = 10) at baseline are shown in Table 1. Patient’s age was 18.3 ± 9.4 years (range 4.7–35.5 years). The biometric *z*-scores indicate reduced somatic growth. Concerning the pathogenetic *MECP2* mutations, half of the patients harbored early truncating mutations. Disease II to IV stages were presented in the examined group while stage I was not found due to the age inclusion criterion (age ≥ 3 years).

**Table 1:** Demographic and clinical features of the examined RTT patients (*n* = 10).

Variables	Mean ± SD Median [IQR] (range)
	N/total
Age (years)	18.3 ± 9.4 (4.7 – 35.5)
Head circumference (*z*-scores^1^)	−1.9 ± 0.8 (−3.0 – 0.0)
Height (*z*-scores)^a^	−1.54 ± 1.39 (−3.69 – 0.04)
Body weight (*z*-scores)^a^	−2.4 [-2.5 – −1.9] (−3.6 – 1.1)
BMI (*z*-scores for age)^a^	−1.3 ± 1.7 (−4.6 – 1.2)
*MECP2 mutation category*	
Early truncating	05/10
Gene deletion	01/10
Late truncating	01/10
Missense	03/10
*Disease stage*	
Stage I	−
Stage II	02/10
Stage III	03/10
Stage IV	05/10
*Disease severity*	
CSS total score	21.3 ± 5.2 (15 – 32)
MBAS total score	51.0 ± 8.1 (32.5 – 61.5)
RSBQ total score	44.8 ± 8.9 (33 – 58)
MPSS total score	58.5 [53.5 – 61.5] (36.5 – 63.5)
MPSS main sub-score	40.2 ± 7.1 (25 – 48)
MPSS supplementary sub-score	15.9 ± 2.1 (11.5 – 18.5)
*Epilepsy*	
Epilepsy history	09/10
Active epilepsy	05/10
General slowing of background EEG	05/10
Multifocal EEG activity	08/10
ASM therapy	8
Mono-therapy	3/8 (37.5)
Multi-therapy	5/8 (62.5)
*Respiratory dysfunction*	
Subclinical hypoxia (SpO_2_ < 90%)	05/10
Apneas (MBAS sub-item)	
25% of time	04/10
50 % of time	03/10
75% of time	02/10
100% of time	01/10
*Hyperventilation (MBAS sub-item)*	
None	05/10
25% of time	03/10
50 % of time	01/10
75% of time	01/10
*Sleep disorders*	
Sleep hours/day	9.9 [8.5 – 10.0] (7.5 – 10.0)
Sleep latency (min)	18.7 ± 16.3
SDSC total score	40.6 ± 7.1 (33.5 – 54.0)
DIMS (SDSC sub-item)	9.5 [7.5 – 12.0] (7.0 – 20.5)
SBD (SDSC sub-item)	5.8 ± 1.4 (3.0 – 7.5)
DA (SDSC sub-item)	3.0 [3.0 – 3.0] (3.0 – 5.5)
SWTD (SDSC sub-item)	9.1 ± 2.7 (6.0 – 14.5)
DOES (SDSC sub-item)	9.0 ± 1.1 (5.0 – 15.0)
SHY (SDSC sub-item)	2.0 [2.0 – 2.0] (2.0 – 8.0)
*Fatigue indirect index*	
Enough energy for daily activities in the last month (QI-Disability sub-item)	
Sometimes	03/10
Often	06/10
Very often	01/10

Qualitative data were described using numbers and percentages. Quantitative data were expressed as mean ± SD or median [inter-quartile range].

a Calculated 𝑧-scores for age are referred to as standard growth charts. BMI, body mass index; CSS, Rett Clinical Severity Score; MBAS, Motor Behavior Assessment Scale; RSBQ, Rett Syndrome Behavior Questionnaire; MPSS, Multi-System Profile of Symptoms Scale; ASM, antiseizure medication; SpO_2_, oxyhemoglobin saturation; SDSC, Sleep Disturbance Scale for Children; DIMS, Disorders of initiating and maintaining sleep; SBD, Sleep breathing disorders; DA, Disorders of arousal; SWTD, Sleep–wake transition disorders; DOES, Disorders of excessive somnolence; SHY, Hyperhydrosis; QI-Disability, Quality of Life Inventory-Disability.

Overall, half of the patients were clinically severe according to disease-specific scores. While approximately half of the patients showed active seizures, a positive history of epilepsy was actually present in the overwhelming majority of the examined RTT patients. Indeed, the majority of RTT patients were on anti-seizure medication (ASM), either as monotherapy or multitherapy. Key RTT-related EEG alterations (i.e., general slowing of background EEG and multifocal EEG activity) were detected in *n* = 5 and *n* = 8 patients, respectively. Respiratory dysfunction was clinically evaluated at baseline visit as subclinical hypoxia and prevalence of apneas and/or hyperventilation during the daytime (i.e., MBAS sub-items). Half of the RTT patients exhibited SpO_2_ values of <90%. Majority of the patients (*n* = 6) exhibited apneas in >50% of daytime, whereas hyperventilation was present in *n* = 4 of RTT patients. Sleep disorders are known to be a key feature of the disease. Indeed, *n* = 6 patients evidenced global alteration of sleep quality (evaluated as SDSC score > 39). Key altered components were represented by DIMS, SBD, SWTD, and DOES. Of note, the fatigue indirect index (as measured as a specific QI-Disability questionnaire sub-item) suggested the presence of a fatigue status in approximately one-third of the patients. Muscle hypotonia (*n* = 7), bruxism (*n* = 9), and facial hypomimia (*n* = 8) were the most prevalent features in the examined RTT group, as well as hypoalgesia (*n* = 9), whereas hypertonia and hyperreflexia were equally distributed (*n* = 5) (Table 2). Half of the patients were non-ambulatory. Scoliosis was present in 6/10 patients (50% with Cobb angle >40°), whereas *n* = 2 patients had surgical correction. Two patients had orthotic devices.

**Table 2:** Motor signs in the examined RTT patients (*n* = 10).

Area	Item	Mean ± SD N/total
	Orthotic devices	02/10
	MBAS III (Motor/physical signs) sub-score	19.3**±** 6.5
Muscle	Muscle hypotonia	07/10
	Tendon retractions	02/10
	Bruxism	09/10
	Dystonia	
	Absent	07/10
	1 joint	02/10
	> 1 joint	01/10
	Hypomimia	
	Absent	02/10
	< 10% of time	08/10
	Hypertonia/rigidity	
	Absent	05/10
	Upper/lower limbs	04/10
	Generalized	01/10
	Hyperreflexia	
	Absent	05/10
	> 2 joints	04/10
	> 2 joints with clonus	01/10
	Myoclonus	
	Absent	08/10
	25% of time	02/10
Perception	Hypoalgesia	09/10
Postural	Walking ability	
	Ambulatory	05/10
	Non-ambulatory	05/10
	Scoliosis	
	No	02/10
	Cobb angle 1° – < 20°	01/10
	Cobb angle 20° – 40°	02/10
	Cobb angle ≥ 40°	03/10
	Surgical correction	02/10
	Kyphosis	01/10
	Oculogyric movements	
	Absent	07/10
	25% of time	01/10
	50% of time	02/10
Functional	Ataxic gait	04/10
	Hand stereotypic hand movements	10/10
	Purposeful hand use	
	Conserved	01/10	
	Partially conserved	03/10
	Lost/never acquired	06/10
	Motor regression	
	No motor ability	04/10
	Hand use	03/10
	Walking ability and use of 1-2 hands	02/10
	Dyspraxia and bilateral pincer grap	01/10
	Parkinson-like tremors	03/10
	Dyskinesia	
	Absent	08/10
	25% of time	02/10
	Truncal rocking	
	Absent	05/10
	25% of time	02/10
	50% of time	03/10
	Fear of movement^1^	02/10
	Bradykinesia	—

Qualitative data were described using numbers and percentages. Quantitative data were expressed as mean ± SD. MBAS, Motor Behavior Assessment Scale; RSBQ, Rett Syndrome Behavior Questionnaire. ^1^ as referred to the corresponding RSBQ sub-item.

### 3.2 Continuous wearable sensor device in-home monitoring

**Figure 1:**
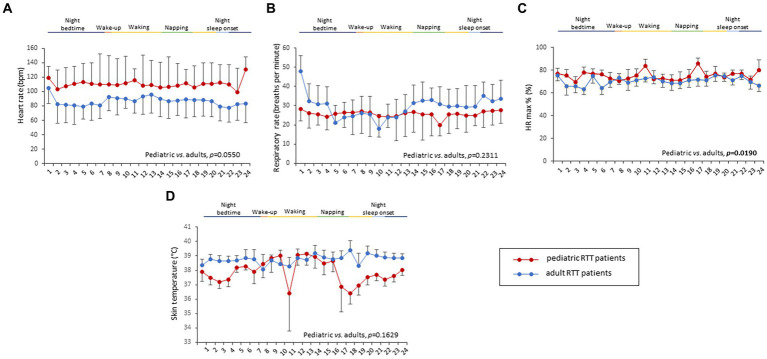
Daytime and night-time variations in biovital parameters as measured by continuous home monitoring of Rett syndrome (RTT) patients remotely. Red color denotes variations for RTT patients with pediatric age and blue color denotes variations for RTT patients with adult age. A significant difference was observed in the percentage of maximum HR [*p* = 0.0021, **(C)**] between the two groups, whereas no significant difference was observed in heart rate **(A)**, respiratory rate **(B)**, and skin temperature **(D)**. Data are expressed as a median and semi-interquartile range; HR, heart rate. Bold characters indicate statistically significant differences.

Overall, a total of 6,559.79 h of bio-vital measures were recorded, of which 77% of the measurements were analyzable. From a post-hoc analysis, discarded signals were predominantly attributed by transient wearability issues. As a result, a total of 5,051.04 h, including 3.176,89 h during the daytime (conventionally 08.00 to 22.00) and 1.874,14 h during the night-time (conventionally 22.00 to 08.00), were analyzed. Individual night sleep hours (median: 10 h, interquartile range: 10 to 10), napping hours (median: 3 h, interquartile range: 2 to 4), and waking hours (mean: 10.9 ± 1.37 h, interquartile range: 9 to 13) were recorded by individual parents/caregivers. In particular, the total recorded biovital measurements corresponded to 2,238 h, 2,121 h, and 692 h for waking, night sleeping, and napping times, respectively. Mean SpO_2_ values at baseline were 81.7 ± 5.0% in hypoxic RTT patients vs. non-hypoxic RTT patients showing values all within the physiological range (96.5 ± 1.1%). No adverse effects were reported during the wearable sensor device recordings, with the one exception of a single episode of transient irritant contact dermatitis that did not require protocol discontinuation. Twenty-four-hour continuous monitoring parameters and environmental monitoring parameters were examined as a function of age category (child and adolescent/adult), disease progression (i.e., clinical stages), and pathogenic *MECP2* mutation category (early truncating, large deletion, late truncating, and missense) (Figure 1 and Table 3). Among the measured biovital parameters, HRmax % was significantly different as a function of age category (*p* = 0.0190) (Figure 1). No significant differences were observed for HR, RR, and skin temperature when comparing pediatric RTT patients to adolescent and/or adult patients (*p* = 0.0550*, p* = 0.2311, *p* = 0.1629, respectively) (Figure 1). Significant age-dependent differences in HRmax % values were detectable in pediatric RTT patients compared to the adolescent/adult group (*p* = 0.0001 and *p* = 0.0190, respectively). A positive significant correlation between mean HR at the clinical visit and mean HR during 24 h monitoring was evidenced (r = 0.7134, *p* = 0.0205), whereas no significant relationship regarding clinically assessed respiratory rate vs. 24 h monitoring evaluation was observed (r = 0.120, *p* = 0.749) (Supplementary Figure S1). As a function of disease progression, only RR and skin temperature values were significantly different in the clinical stages (i.e., II, III, and IV) (*p* ≤ 0.0082) (Table 3). All the biovital parameters were significantly associated with the *MECP2* mutation category (*p* ≤ 0.002) except for HR value (*p* = 0.3374) (Table 3). No statistically differences were observed as a function of clinical severity (i.e., CSS, MBAS, RSBQ, and SDSC) (data not shown).

**Table 3:** 24-h continuous home monitoring in RTT patients (*n* = 10) as a function of clinical stage and *MECP2* mutation category.

Variable		Clinical stage			*p* value	*MECP2* mutation category				*p*-value
		II (A)	III (B)	IV (C)		Early trunc. (A)	Large Del. (B)	Late trunc. (C)	Missense (D)	
Wearable monitoring parameters	HR (bpm)	108.6 [33−180.7]	88 [65.6−147]	90.2 [56.7−141.5]	0.686	102 [49.7−160]	95 [74.6−116.2]	73.0 [48.2−153]	100.5 [56.7−148.2]	0.3374
	RR (breaths per min)	26.2 [12−55]	18.4 [10−33]	32.8 [12−53]	**<0.0001**	27.1 [12−51]	15.3 [10−21]	18.4 [7.2−34.7]	26.5 [12−53.2]	**<0.0001**
	HR max % (%)	71.4 [58.3−80.4]	75.3 [63−82.5]	71.7 [61.6−82.1]	0.6231	71.6 [60−80.5]	76.9 [65.3−85.0]	65.5 [53.5−75.9]	74.6 [64.1−85.4]	**0.0001**
	Skin Temperature (°C)	38.6 [38.6−39.2]	38.3 [37.4−39.4]	38.0 [35.9−39.1]	**0.0082**	37.35 [35.9−39.2]	32.7 [27.6−36.1]	39.3 [38.4−39.5]	38.7 [37.2−39.0]	**<0.0001**
HRV time−domain parameters	RMSSD (ms)	300.1 [18.5−2021.7]	179.9 [59−341]	148 [25.2−566.9]	**0.0004**	246.6 [49.7−608.9]	61.5 [51.6−107.1]	90.9 [12.2−265.4]	166.4 [11.6−1334.6]	**0.0004**
	SDNN (ms)	228.8 [21.2−1409.7]	138.9 [49.7−250.8]	104.9 [16.8−741.9]	**0.0004**	183.2 [37.8−550.6]	73.2 [61.101.8]	95.7 [15.5−240.1]	130.8 [12.3−924]	**0.0005**
	SDRR (ms)	27.9 [3.12−69.2]	22.5 [4.8−48.3]	12.4 [1.3−42.4]	**<0.0001**	24.8 [3.2−53.7]	6.9 [5.9−13.0]	9.3 [1.0−47.8]	18.7 [1.5−43.4]	**0.0183**
	CVSD (%)	0.45 [0.02−2.03]	0.30 [0.06−0.62]	0.17 [0.02−1.24]	**<0.0001**	0.33 [0.05−1.1]	0.15 [0.02−0.72]	0.15 [0.01−0.34]	0.25 [0.02−1.53]	**0.0004**
	pNNI−20 (%)	70.9 [20.3−100]	77.3 [32.3−96.5]	54.7 [9.1−100]	**0.0138**	75.4 [44.1−100]	67.4 [60−77.3]	45.2 [4.9−88.7]	56.5 [2.8−100]	**0.0008**
	pNNI−50 (%)	53.2 [0−100]	51.3 [12.1−86.9]	26.4 [0−81.7]	**0.0079**	54.7 [13.6−94.4]	35.5 [26.8−40.7]	19.24 [0−62.1]	34.5 [0−82.8]	**0.0005**
	SDSD (ms)	292.5 [15.5−1990.3]	173.2 [45.0−333.4]	123.5 [17.6−284.7]	**0.0002**	210.3 [39.1−745.8]	61.5 [51.6−106.9]	85.6 [11.5−258.8]	157.7 [10.7−1324]	**0.001**
	M−NNI (ms)	732.8 [407.1−1117.1]	492.4 [413.7−835.2]	545.2 [413.9−925.8]	**0.0042**	551.3 [421.2−1008.4]	681.5 [471.1−805.8]	479.7 [385.6−1024.7]	561.2 [403.8−936.6]	0.3359
	CVNNI (%)	0.34 [0.03−1.45]	0.24 [0.06−0.44]	0.125 [0.02−0.33]	**<0.0001**	0.28 [0.04−0.81]	0.09 [0.08−0.15]	0.11 [0.02−0.31]	0.11 [0.02−0.32]	**<0.0001**
HRV frequency−domain parameters	LFnu (Hz)	18.5 [17.7−18.7]	14,203.00 [19.8 −15,294.0]	18.0 [16.5−103.6]	**<0.0001**	18.7 [17.0 −12,711.0]	N.A.	15,151.0 [14,321.0−15,447.0]	18.2 [17.3−18.6]	**<0.0001**
	HFnu (Hz)	81.5 [80.6 −82.3]	85.7 [84.7 −80,149.0]	83.1 [81.7−84.2]	**<0.0001**	83.7 [81.9 −85.0]	N.A.	84.8 [84.5−85.6]	81.8 [81.4−82.7]	**<0.0001**
	LF / HF ratio	0.229 [0.218−0.232]	0.185 [0.173 −0.246]	0.205 [0.190−0.225]	**<0.0001**	0.214 [0.193−0.235]	N.A.	0.181 [0.171−0.184]	0.224 [0.212−0.230]	**<0.0001**
	Total power (ms^2^)	379.3 [248.9−578.8]	37.1 [22.5−478.4]	95.5 [22.9−243.9]	**<0.0001**	309.6 [90.2−553.1]	N.A.	25.8 [16.9−35.5]	176.5 [10.9−278.5]	**<0.0001**
Indoor air (IA) monitoring parameters	IA CO_2_ (ppm)	744.2 [407−1346]	1095.5 [760.2−1528]	808.1 [402−1495]	**<0.0001**	887.5 [458.5−1493]	N.A.	1210.7 [955.5−1634.5]	711.1 [386.5−1418]	**<0.0001**
	IA relative humidity (%)	65 [55−74]	65.6 [53−72]	67.3 [52−70]	0.1569	65 [55−74]	N.A.	63.6 [53−72]	67.3 [52−70]	0.1569
	IA noise level (dB)	40.1 [33−66]	35.5 [32−48]	42.2 [34−73]	0.2377	38.6 [33−50]	N.A.	37.3 [32−48]	39.0 [32−58]	0.4203
	IA atmospheric pressure (mbar)	1015.1 [1014.4−1021.2]	1014.4 [1013−1015.5]	1016.4 [1013.3−1020.2]	**<0.0001**	1015.2 [1013.4−1020]	N.A.	1014.4 [1013.9−1017.5]	1014.7 [1013.7−1018]	**0.0158**
	IA temperature (°C)	22.36 [20.6−27.1]	19.62 [19.2−23.8]	22.9 [20−25.3]	**<0.0001**	21.4 [19.5−25.1]	N.A.	22.0 [18.8−23.7]	22.9 [19.1−25.5]	0.1484
	P_H2O_ (pt) (mbar)	6.8 [6.4−7.2]	6.5 [6.3−9.7]	7.4 [5.2−8.4]	0.228	7.7 [6.5−9.6]	N.A.	6.4 [6.3−9.5]	6.7 [3.3−7.1]	**<0.0001**
	P_CO2_ (pt) (ppm)	313.5 [243.1−447.8]	693.0 [211.3−885.8]	388.1 [214.7−585.3]	**<0.0001**	391.0 [242.0−594.6]	N.A.	790.7 [713.2−905.3]	257.7 [206.7−484.3]	**<0.0001**

HR, heart rate; RR, respiratory rate; HRmax %, percentage of maximum heart rate; Skin Temp, skin temperature; RMSSD, Root Mean square of successive RR interval differences; RR intervals, interbeat intervals between all successive heartbeats; SDNN, Standard Deviation of all NN intervals; NN intervals, interbeat intervals with artifacts removed; SDRR, standard deviation of RR intervals; CVSD, RMSSD divided by Mean NNI; pNNI-20, Percentage of successive RR intervals differing more than 20 ms; pNNI-50, Percentage of successive RR intervals differing more than 50 ms; SDSD, SD of successive differences between NN; M-NNI, Mean of NN; CVNNI, SDNN divided by mean NN; LFnu, normalized Low-Frequency power; HFnu, normalized High-Frequency power; IA, indoor air; P_H2O_ (pt), water vapor partial pressure in the bedroom (patient’s contribution); P_CO2_ (pt), CO_2_ partial pressure in the bedroom (patient’s contribution); NA, data not available; A.U.; arbitrary units. *Post-hoc analysis clinical stage*: A > B ≈ C: RMSSD, SDNN, SDRR, CVSD, SDSD, M-NNI; A > B > C: CVNNI; A > C: pNNI-20, pNNI-50; B < A ≈ C: RR, IA atmospheric pressure, IA temperature; B > A ≈ C: IA CO_2_; C < B ≈ A: Skin Temp; B > A ≈ C: P_CO2_ (pt). *Post-hoc analysis MECP2 mutation category*: A > B ≈ C: RMSSD, SDNN, SDRR, CVSD, SDSD, M-NNI; A > B > C: CVNNI; A > C:pNNI-20, pNNI-50; B < A ≈ C: RR, IA atmospheric pressure, IA temperature; B > A ≈ C: IA CO_2_; C < B ≈ A: Skin Temp, A > B ≈ C: P_H2O_ (pt), B > A > C: P_CO2_ (pt). Bold character indicates significant differences.

### 3.3 HRV time-domain metrics

**Figure 2:**
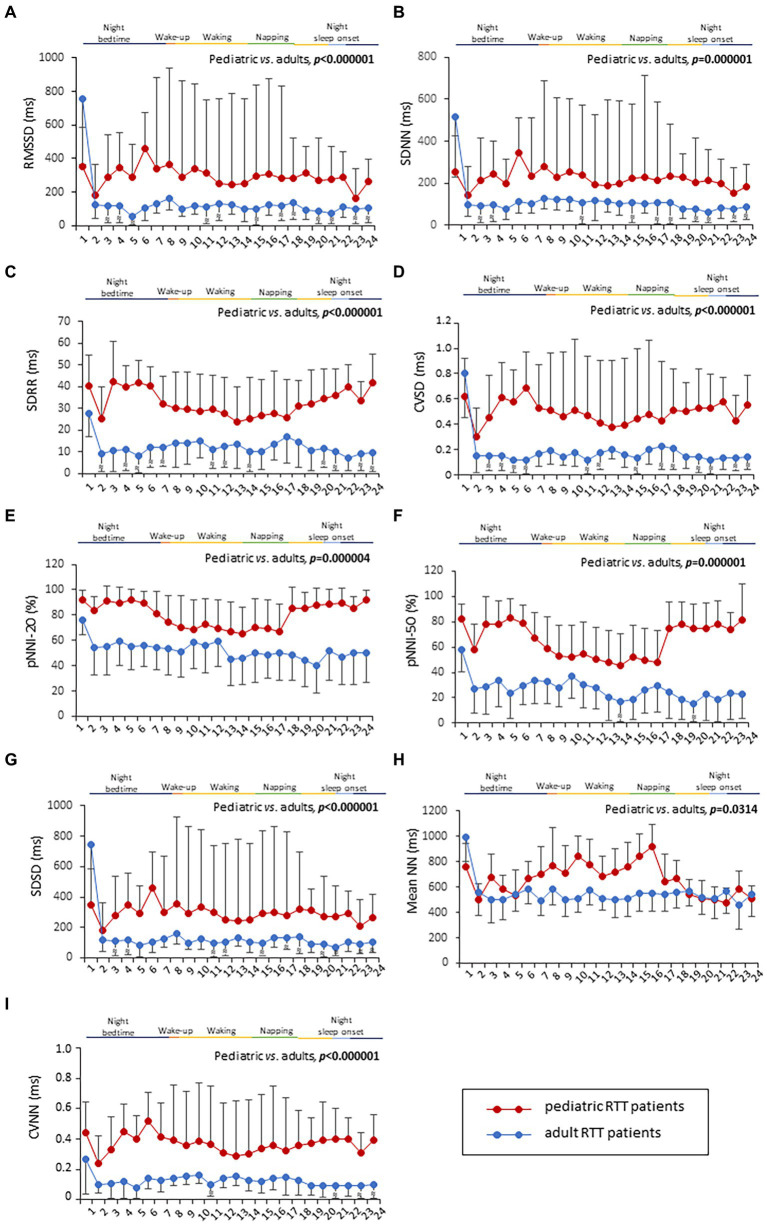
Twenty-four-hour trends in time-domain parameters of heart rate. All the examined parameters (RMSSD, SDNN, SDRR, CVSD, pNNI-20, pNNI-50, SDSD, Mean NN, and CVNN) were statistically different between pediatric RTT patients (in red color) and adolescent/adult RTT (in blue color) **(A–I)**. RMSSD, Root Mean square of successive R-R interval differences; R-R intervals, interbeat intervals between all successive heartbeats; SDNN, Standard Deviation of all NN intervals; NN intervals, interbeat intervals from which artifacts have been removed; SDRR, standard deviation of RR intervals; CVSD, RMSSD to MeanNNI ratio; pNNI-20, Percentage of successive R-R intervals that differ by more than 20 ms; pNNI-50, Percentage of successive R-R intervals that differ by more than 50 ms; SDSD, SD of successive differences between NN; M-NNI, Mean of NN; CVNNI, SDNN divided by mean NN. Bold characters indicate statistically significant differences.

HRV time-domain metrics during the 24 h continuous home monitoring are shown in Figure 2. Although partially overlapping at 1:00 AM, Root Mean square of successive interbeat intervals between all successive heartbeats (R-R) differences (RMSSD), Standard Deviation of all (NN) (SDNN) interbeat intervals with artifacts removed, CVSD (RMSSD divided by Mean NNI), and SDSD (SD of successive differences between NN) were increased in RTT of pediatric age as compared to those measured in adolescent/adult RTT patients (RMSSD: *p* < 0.000001, SDNN: *p* = 0.000001, CVSD: *p* < 0.000001, SDSD: *p* < 0.000001) (Figure 2).

In pediatric RTT patients, SDRR (standard deviation of R-R intervals), pNNI-20 (percentage of successive RR intervals differing more than 20 ms), pNNI-50 (percentage of successive RR intervals differing more than 50 ms), and CVNN (SDNN divided by mean NN) showed distinct increased patterns when compared to RTT of adolescent/adult age (SDRR: *p* < 0.000001, pNNI-20: *p* = 0.000004, pNNI-50: *p* = 0.000001, CVNN: *p* < 0.000001) (Figure 2). The only examined variable that exhibited some partially overlapping periods (i.e., night bedtime, napping, and night sleep initiation) was represented by mean NN (*p* = 0.0314). For the HRV time-domain parameters, all the examined variables were significantly different in all disease stages (*p* ≤ 0.0138) (Table 3). With a single exception of M-NNI, significant differences for the HRV time-domain parameters in all the *MECP2* mutation category groups were evidenced (*p* ≤ 0.0183) (Table 3). Conversely, no statistical differences as a function of disease severity scores (i.e., CSS, MBAS, RSBQ, and SDSC) were observed (data not shown).

### 3.4 HRV frequency-domain metrics

**Figure 3:**
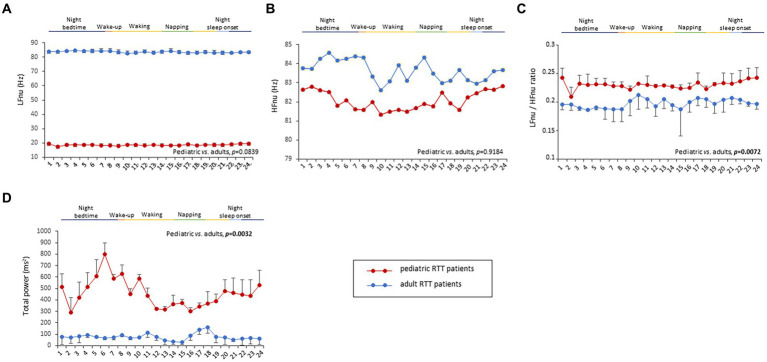
Twenty-four-hour trends in time-domain parameters of heart rate variability. No statistically significant difference was found for LFnu **(A)** and HFnu **(B)** when compared to RTT of pediatric age (red color) and RTT of adolescent/adult age (blue color). Statistically significant differences were evidenced in LFnu/HFnu ratio [*p* = 0.0072, **(C)**] and total spectral power [*p* = 0.0032, **(D)**]. LFnu, normalized Low-Frequency power; HFnu, normalized High-Frequency power. Bold characters indicate significant differences.

To understand the possible influence of sympathovagal imbalance, the HRV frequency-domain parameters were also evaluated (Figure 3). Although adolescent/adult RTT patients showed apparently increased values in LFnu and HFnu, no significant difference was observed as compared with pediatric patients (*p* = 0.0839 and *p* = 0.9184, respectively). Conversely, significantly increased LFnu/HFnu ratio and total spectral power were evidenced in pediatric RTT patients as compared to the adolescent/adult patient counterpart (*p* = 0.0072 and *p* = 0.0032, respectively). When examining *the MECP2* mutation category, despite missing data for the large deletion group, the HRV frequency-domain metrics were significantly different (*p* < 0.0001) (Table 3). On the contrary, no statistically significant differences as a function of disease severity scores (i.e., CSS, MBAS, RSBQ, and SDSC) were evidenced (data not shown).

### 3.5 Environmental parameters monitoring

**Figure 4:**
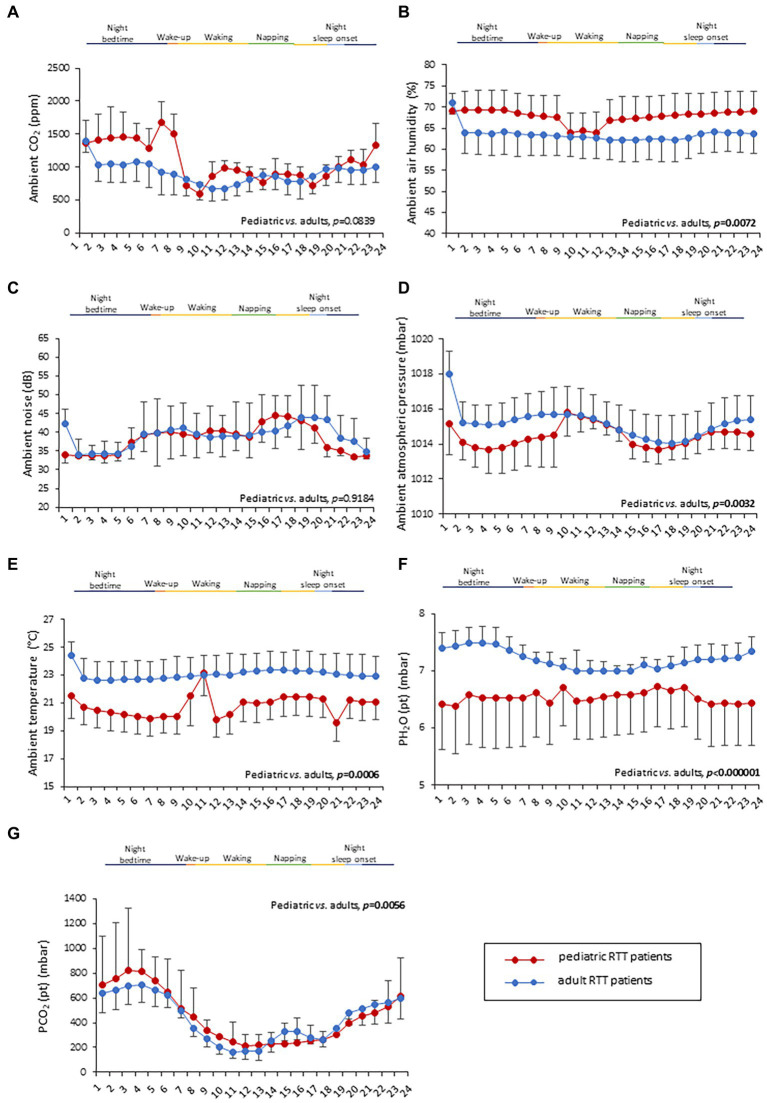
Twenty-four-hour variation of indoor air quality parameters measured in the bedroom of RTT patients. Variations in pediatric RTT patients and adolescent/adult patients are shown in red and blue colors, respectively. Significant differences were observed in air humidity [*p* = 0.0072, **(B)**], atmospheric pressure [*p* = 0.0032, **(D)**], and air temperature [*p* = 0.0006, **(E)**]. Conversely, no significant differences were observed for CO_2_ levels **(A)** and noise **(C)**. Significant differences were also observed for both patient-derived parameters, P_H2O_ (pt) [*p* < 0.000001, **(F)**] and P_CO2_ (pt) [*p* < 0.000001, **(G)**]. Data are expressed as a median and semi-interquartile range. Statistically significant differences are indicated in bold character. P_H2O_ (pt), water vapor partial pressure originating from patients in the bedroom; P_CO2_ (pt), CO_2_ partial pressure originating from patients in the bedroom.

No statistically significant difference was observed in CO_2_ levels and ambient noise levels between the pediatric and adolescent/adult RTT groups (*p* = 0.0839 and *p* = 0.9184, respectively) (Figures 4A–E). Significantly increased air humidity was observed in the environment of the pediatric RTT patients when compared to the adolescent/adult counterpart (*p* = 0.0072), albeit with a minimal overlapping time period (waking). Although ambient atmospheric pressure showed similar trends in both groups, increased values were shown in adolescent/adult RTT patients (*p* = 0.0032) and ambient temperature (*p* = 0.0006) (Figures 4A–E). Moreover, significant differences were evidenced in CO_2_, atmospheric pressure, and temperature values (*p* ≤ 0.0001) with disease stage. Significant differences were observed in the CO_2_ and atmospheric pressure when examining the *MECP2* mutation type (*p* ≤ 0.0158), while no data were available for the large deletion mutation group (Table 3). The possible contribution of the environmental variables on clinical severity scores in RTT patients was evaluated and no statistically significant differences for CSS, MBAS, RSBQ, and SDSC scoring were observed (data not shown).

### 3.6 Patient-derived environmental parameters monitoring

Two indirectly estimated patient-derived parameters, i.e., the patient contribution to CO_2_ partial pressure [P_CO2_ (pt)] and H_2_O partial pressure [P_H2O_ (pt)], were also examined in this study. Patients’ contributions to H_2_O and CO_2_ molar densities, as well as their heat fluxes, were estimated by applying Eqs 13–15. Time-resolved estimations of these parameters exhibited complex mutual behaviors and complex patterns in the scatterplots with various biophysical parameters. An increase in water vapor emitted by a given patient can be sensed by the environment sensors with a possible delay, depending on variables influencing the air circulationin the room. This delay phenomenon hinders a time-resolved comparison among environmental signals originating from patient-derived molecules with differences in diffusivity and/or molecular weight, such as H_2_O and CO_2_. Therefore, we have investigated possible correlations among the time averages of biophysical and environmental parameters, obtaining results summarized in Supplementary Figure S2.

This figure (Supplementary Figure S2) shows selected examples of the relationship (scatterplots) among data extrapolated from environmental sensors and biophysical parameters (HR and RR). All data have been averaged over time, with each point in the three images representing data from a single patient. In addition, the data shown in these plots represent a partial indirect validation of the physical/mathematical modeling applied for estimating the patient parameters from the collected environmental data.

The time average of patient’s contribution to water vapor molar density ${\bar{\rho }}_{H2O}$ shows a good linear correlation with the average heat flux $\bar{\dot{Q}}$ ( $R^{2}=0.8235$ ) and CO_2_ molar density ${\bar{\rho }}_{CO2}$ ( $R^{2}=0.7566$ ). Moreover, ${\bar{\rho }}_{H2O}$ and $\bar{\dot{Q}}$ showed robust correlations with several biophysical parameters. The heat flux $\bar{\dot{Q}}$ had a strong linear correlation with the HR ( $R^{2}=0.9385$ ) and with a power relationship ( $\bar{NNI}≅3345.8\cdot {\bar{\dot{Q}}}^{-0.727}$ ) of an average $\bar{NNI}$ ( $R^{2}=0.9244$ ).

Interestingly, both the patient-derived variables resulted in a statistical difference in RTT patients as a function of age category (Figures 4F,G). In particular, RTT of adolescent/adult age showed statistically significant increase in P_H2O_ (pt) levels when compared to pediatric RTT patients (*p* < 0.000001) (Figure 4F). The pediatric population exhibited increased values in P_CO2_ (pt) during the night bedtime and first half of the day, whereas the adolescent/adult patients showed higher values in the second half of the day (*p* = 0.0056) (Figure 4G). Furthermore, a significant difference was observed in P_CO2_ (pt) as a function of clinical stages of the disease (*p* ≤ 0.0001) (Table 3). Moreover, both P_CO2_ (pt) and P_H2O_ (pt) values differed as a function of the *MECP2* mutation category (*p* < 0.0001) (Table 3).

Supplementary Figure S2 shows that ${\bar{\rho }}_{H2O}$ has a high linear correlation ( $R^{2}=0.9528$ ) with the mean HR values, and that the patients’ contribution to the H_2_O partial pressure ${\bar{P}}_{H2O}\left(pat\right)\;$ using Eq. 6, is strongly correlated with the average breath rate values. Supplementary Figure S2A shows selected examples of relationships (scatterplots) among data extrapolated from environmental sensors and biophysical parameters (HR and RR). All data were averaged over time where each point in the three images represents data from a single patient. Data shown in these plots are also an indirect partial validation of the physical/mathematical modeling adopted for estimating patient parameters from the collected environmental data. Panel A illustrates the patient contribution to water vapor relative molar density (per body mass unit) vs. the estimated flux of heat lost by the patient due to the patient–environment temperature difference. The heat flux was estimated using skin temperature data collected by the wearable t-shirt, the environment temperature acquired by the meteorological station, and the patient’s height and mass. The corresponding water vapor molar density stemming from the relative humidity measured by the meteorological station was estimated. Although the interpretation is complex, the evidenced pattern confirms the existence of a clear relationship between the data.

Supplementary Figure S2A sketches the relationship between patient heat loss and patient’s contribution to water vapor relative molar density (per body mass unit). The patient’s contribution to the water vapor molar density (per body mass unit) has been estimated to be derived from the relative humidity measured by the meteorological station. Supplementary Figure S2B plot also confirms the existence of a strong relationship between these data, which can be interpreted by the augmentation in the heart rate, implying an increase of the emitted water vapor. Finally, Supplementary Figure S2C shows the relationship between RR and the patient’s contribution to water vapor partial pressure, estimated from the relative humidity measured by the weather station with indoor sensors. The scatterplot confirms the existence of a relationship between these data, which can be interpreted by the augmentation in the respiratory rate related to an increase in the emitted water vapor.

The possible contribution of the two patient-derived parameters, [i.e., P_CO2_ (pt) and P_H2O_ (pt)], on clinical severity scores in RTT patients was evaluated. Interestingly, significant relationships were evidenced between the MPSS scale sub-items (either mean MPSS or MPSS supplement) and P_H2O_ (pt) values (Supplementary Figure S3). In particular, for the MPSS mean scale, the most relevant negative significant correlations were found with mental health (r = −0.481, *p* < 0.001), autonomic (r = −0.450, *p* < 0.001), orofacial (r = −0.451, *p* < 0.001), respiratory (r = −0.481, *p* < 0.001), respiratory problems (r = −0.642, *p* < 0.001), and sleep problems sub-scores (r = −0.540, *p* < 0.001) (Supplementary Figure S3). For the MPSS supplement, statistically significant inverse associations with the endocrinology (r = −0.433, *p* < 0.001) and skeletal problems subscores were evidenced (r = −0.630, *p* < 0.001).

### 3.7 Effect of sleep–wake cycle on continuous wearable sensor device and environmental variables

All the 24-h continuous wearable sensor devices and environmental parameters were then examined as a function of the sleep–wake cycle based on caregiver diaries (Figures 1–3). No statistically significant differences were observed in the wearable parameters for all RTT patients (pediatric age *p* ≥ 0.4254 and adolescent/adult age *p* ≥ 0.1674; data not shown). Similarly, no significant difference was found in all the examined HRV time-domain variables for all RTT patients (pediatric age *p* ≥ 0.5324 and adolescent/adult age *p* ≥ 0.3129). Significant differences were evidenced for HRV frequency-domain parameters. In particular, pediatric RTT patients showed significant differences in HFnu and total power values (*p* ≤ 0.0431), while adolescent/adult RTT patients showed significant differences in LFnu values, HFnu values, and LF/HF ratio (*p* ≤ 0.0320). For the IA monitoring variables, we observed statistically significant differences in noise levels and P_CO2_ (pt) values in both pediatric (*p* < 0.001) and adolescent/adult RTT patients (noise levels *p* = 0.0024, P_CO2_ (pt): *p* < 0.0001). Moreover, CO_2_ levels were found to be statistically different in pediatric RTT patients as a function of the sleep–wake cycle (*p* = 0.0001), whereas atmospheric pressure and P_H2O_ (pt) values resulted statistically different in RTT patients of adolescent/adult age (atmospheric pressure *p* = 0.0255; P_H2O_ (pt) *p =* 0.0063).

### 3.8 Effect of clinical sleep disorder (CSD) on continuous wearable sensor device parameters and environmental variables

All parameters of the wearable sensor device were evaluated for clinical sleep disorder (CSD) (Table 4). All the wearable monitoring parameters were evaluated as a function of clinical sleep disorder (CSD) (Table 4). Pediatric RTT patients with CSD exhibited increased values in SDRR, CVSD, pNNI-20, pNNI-50, and SDSD (*p* ≤ 0.032) compared to RTT without CSD. Similarly, all the HRV frequency-domain variables were significantly increased in pediatric RTT patients with CSD (*p* ≤ 0.0008). Concerning the IA monitoring, increased values in atmospheric pressure (*p* = 0.0290) and reduced values in relative humidity (*p* = 0.0360) and P_H2O_ (pt) values (*p* < 0.0001) were evidenced in pediatric RTT patients with CSD (Table 4).

**Table 4:** 24-h continuous home monitoring in RTT patients (*n* = 10) as a function of clinical sleep disorders.

Variable		Clinical Sleep disorders					
		Patients with pediatric age (*n* = 3)		*P*-value	Patients with adolescent/adult age (*n* = 7)		*p*-value
		No (*n* = 1)	Yes (*n* = 2)		No (*n* = 5)	Yes (*n* = 2)	
Wearable monitoring parameters	HR (bpm)	110.6 [34.7–173]	110.8 [65.5–160.5]	0.6874	86.1 [58–133.5]	82.9 [49–157]	0.4766
	RR (breaths per min)	26.33 [12–53.7]	24.6 [11.5–39.5]	0.2163	26.33 [11.0–50.2]	32.5 [14–45.9]	0.6032
	HR max % (%)	74.6 [61.1–85.6]	76.6 [66.7–82.3]	0.8280	73.1 [62.1–83.9]	68.6 [56.9–77.5]	**0.0071**
	Skin Temperature (°C)	38.4 [36.7–38.8]	37.7 [36.4–39.2]	0.3524	38.2 [37.1–39.2]	38.9 [38.2–39.4]	**0.0123**
HRV time-domain parameters	RMSSD (ms)	206.7 [13.4–1980.6]	323.9 [128.5–645.8]	0.067	103.8 [25.2–293.2]	161.7 [12.3–359.8]	0.1882
	SDNN (ms)	149.3 [13.9–1379.2]	237.5 [101.6–468.2]	0.069	86.3 [22.7–238]	130.2 [15.5–274.4]	0.2769
	SDRR (ms)	20.1 [2.5–66.7]	37.5 [11.3–60.5]	**0.032**	10.2 [1.34–34.7]	16.9 [2.3–54.3]	0.1480
	CVSD (%)	0.29 [0.02–1.95]	0.55 [0.18–1.028]	**0.037**	0.14 [0.02–0.475]	0.21 [0.02–0.64]	0.2514
	pNNI-20 (%)	59.17 [8.99–100]	89.1 [68.5–100]	**0.0016**	50.0 [11.1–93.3]	57.3 [6.1–100]	0.054
	pNNI-50 (%)	37.7 [0–100]	78.3 [47.6–100]	**0.0099**	22.13 [0–75]	35.7 [0–86.4]	0.7007
	SDSD (ms)	189.2 [13.6–1974.4]	321.4 [124.5–640.8]	**0.0071**	100.5 [17.2–290.4]	147.1 [11.9–358.2]	0.2929
	M-NNI (ms)	594.3 [413.7–1109.1]	638.8 [414.9–1004.5]	0.9624	526.1 [417.5–901.3]	554.7 [403.7–1011.1]	0.6145
	CVNNI (%)	0.23 [0.02–1.43]	0.4 [0.16–0.71]	0.0510	0.125 [0.02–0.34]	0.110 [0.02–0.027]	0.1556
HRV frequency-domain parameters	LFnu (Hz)	18.5 [17.4–18.6]	18.67 [18.1–19.6]	**<0.0001**	18.1 [16.3–14566.5]	700.6 [17.9–15151.0]	**0.0001**
	HFnu (Hz)	81.5 [81.3–82.6]	82.1 [81.4–80149.0]	**0.0008**	83.6 [82.0–84.3]	84.3 [82.1–87.8]	**0.0052**
	LF / HF ratio	0.230 [0.213–0.231]	0.232 [0.223–0.246]	**<0.0001**	0.197 [0.187–0.221]	0.187 [0.181–0.221]	**0.0054**
	Total power (ms^2^)	279.9 [152.5–392.5]	549.1 [435.2–664.2]	**<0.0001**	73.4 [10.9–142.3]	90.4 [25.8–338.1]	**0.0012**
Indoor air (IA) monitoring parameters	IA CO_2_ (ppm)	933.1 [377–1,650]	959 [662.7–1444.2]	0.2607	818.7 [395.7–1431.5]	968 [598.8–1468.5]	**0.0045**
	IA relative humidity (%)	68.2 [56.5–80]	66 [54.7–74]	**0.0360**	64.3 [58–74.6]	58.3 [40.5–68]	**<0.0001**
	IA noise level (dB)	37.9 [32–49.9]	38.4 [33.9–44.2]	0.2377	39.8 [32.4–53.6]	37.7 [32–55.5]	0.2270
	IA atmospheric pressure (mbar)	1014.4 [1014.0–1021.3]	1014.8 [1012.6–1015.6]	**0.0290**	1015.6 [1013.3–1019.8]	1015.2 [1014.3–1018.5]	0.7263
	IA temperature (°C)	22.9 [15.8–27.1]	21.4 [19.3–23.7]	0.7877	22.9 [19.9–25.3]	21.4 [18.5–24.8]	**0.0330**
	P_H2O_ (pt) (mbar)	7.2 [6.9–7.4]	6.4 [6.3–6.5]	**<0.0001**	8.4 [7.4–9.7]	5.2 [2.4–6.4]	**<0.0001**
	P_CO2_ (pt) (ppm)	406.3 [243.2–629.4]	382.5 [285.7–864.5]	0.1126	400.9 [221.7–585.3]	508.3 [178.9–790.7]	0.066

HR, heart rate; RR, respiratory rate; HRmax %, percentage of maximum heart rate; Skin Temp, skin temperature; RMSSD, Root Mean square of successive RR interval differences; RR intervals, interbeat intervals between all successive heartbeats; SDNN, Standard Deviation of all NN intervals; NN intervals, interbeat intervals with artifacts removed; SDRR, standard deviation of RR intervals; CVSD, RMSSD divided by Mean NNI; pNNI-20, Percentage of successive RR intervals differing more than 20 ms; pNNI-50, Percentage of successive RR intervals that differing more than 50 ms; SDSD, SD of successive differences between NN; M-NNI, Mean of NN; CVNNI, SDNN divided by mean NN; LFnu, normalized Low-Frequency power; HFnu, normalized High-Frequency power; IA, indoor air; PH2O (pt), water vapor partial pressure in the bedroom (patient’s contribution); PCO2 (pt), CO_2_ partial pressure in the bedroom (patient’s contribution); A.U.; arbitrary units. Bold character indicates significant differences.

Adolescent/adult RTT patients with CSD showed significantly reduced HRmax % (*p* = 0.0071) and increased skin temperature values (*p* = 0.0123) compared to adolescent/adult RTT patients without CSD. No statistically significant differences in HRV time-domain parameters (*p* ≥ 0.054) were evidenced in adolescent/adult RTT patients with CSD compared to those without CSD. For the HRV frequency-domain variables, similarly to pediatric RTT patients with CSD, we found increased values in all the parameters of adolescent/adult RTT patients with CSD except for LF/HF ratio that resulted in reduced values (*p* = 0.0054) (Table 5). For the environmental monitoring, although overlapping significant trends with pediatric RTT patients with CSD were evidenced for relative humidity and P_H2O_ (pt) values (*p* < 0.0001), increased CO_2_ levels (*p* = 0.0045) and lower temperature (*p* = 0.0330) were also observed.

**Table 5:** 24-h continuous home monitoring of RTT patients (*n* = 10) as a function of EEG multifocal epileptiform activity and severe general background slowing.

Variable		EEG multifocal epileptiform activity		*P*-value	Severe EEG general background slowing		*P*-value
		No (*n* = 6)	Yes (*n* = 4)		No (*n* = 5)	Yes (*n* = 5)	
Wearable monitoring parameters	HR (bpm)	88.9 [54.7–149.5]	97.6 [51–156]	0.7973	101.7 [51–152]	90.81 [64–152]	0.8857
	RR (breaths per min)	20.6 [13.2–45.5]	26.3 [12–51]	0.6323	26.7 [102–53]	19.8 [12–38.7]	**0.012**
	HR max % (%)	69.0 [58.2–80.4]	73.7 [62–86.7]	**0.0076**	71.5 [60.4–81.4]	74.1 [63–82.5]	0.5018
	Skin Temperature (°C)	38.2 [37.5–39.3]	38.0 [36.5–39.2]	**0.0120**	38.48 [36.7–39.1]	38.36 [37.1–39.4]	**0.0279**
HRV time-domain parameters	RMSSD (ms)	139.7 [13.2–344.7]	188.4 [34.2–625.6]	0.0670	205.2 [25.2–1,242]	129.2 [22.7–373.9]	**0.0227**
	SDNN (ms)	139.6 [26.3–550.6]	117.4 [15.8–256.6]	0.1457	151.63 [22.3–916.1]	108.7 [21.5–281.7]	0.0823
	SDRR (ms)	18.4 [2.7–49.7]	13.6 [1.3–53.4]	0.1350	10.2 [1.34–34.7]	16.9 [2.3–54.3]	0.4696
	CVSD (%)	0.25 [0.02–0.52]	0.17 [0.02–0.52]	0.0670	0.14 [0.02–0.47]	0.21 [0.02–0.64]	0.0590
	pNNI-20 (%)	68.2 [25.2–100]	57.7 [6.1–100]	0.0740	50.0 [11.1–93.3]	57.3 [6.1–100]	0.8632
	pNNI-50 (%)	22.13 [0–75]	35.7 [0–86.4]	0.0660	22.1 [0–75]	35.7 [0–86.4]	0.8048
	SDSD (ms)	100.5 [17.2–290.4]	147.1 [11.9–358.2]	0.0870	100.5 [17.2–290.4]	147.1 [11.9–358.2]	**0.0470**
	M-NNI (ms)	549 [414.6–976.5]	559.6 [403.7–1005.3]	0.5479	526.1 [417.5–901.3]	554.7 [403.7–1011.1]	**0.0270**
	CVNNI (%)	0.11 [0.03–0.81]	0.21 [0.02–0.27]	**<0.0001**	0.12 [0.02–0.34]	0.11 [0.02–0.27]	0.7736
HRV frequency-domain parameters	LFnu (Hz)	700.6 [17.9–15151.0]	18.5 [17.2–19.7]	**<0.0001**	700.6 [17.9–15151.0]	18.5 [17.2–19.7]	0.7819
	HFnu (Hz)	84.3 [82.0–84.8]	82.6 [81.5–84.3]	**<0.0001**	81.9 [81.4–82.9]	84.6 [82.6–86.2]	**<0.0001**
	LF / HF ratio	0.187 [0.181–0.221]	0.220 [0.196–0.231]	**<0.0001**	0.223 [0.208–0.231]	0.195 [0.183–0.207]	**<0.0001**
	Total power (ms^2^)	90.4 [25.8–250.3]	191.1 [70.1–475.3]	**<0.0001**	243.9 [97.8–379.3]	66.1 [32.0–158.3]	**<0.0001**
Indoor air (IA) monitoring parameters	IA CO_2_ (ppm)	941.6 [476.2–1,529]	933 [477.3–1348.8]	0.2956	746.5 [402–1,322]	1,047 [610–1,616]	**<0.0001**
	IA relative humidity (%)	68.29 [56–74]	61.6 [38.2–69]	**<0.0001**	64.3 [58–74.6]	58.3 [40.5–68]	**<0.0001**
	IA noise level (dB)	39.8 [32.4–53.6]	37.7 [32–55.5]	0.2270	39.8 [32.4–53.6]	37.7 [32–55.5]	0.2270
	IA atmospheric pressure (mbar)	1014.8 [1013.3–1019.8]	1015.2 [1014.3–1018.5]	0.1182	1015.6 [1013.3–1019.8]	1015.2 [1014.3–1018.5]	0.7263
	IA temperature (°C)	21.5 [19.5–22.9]	22.9 [18.6–25.1]	0.5141	22.9 [19.9–25.3]	21.4 [18.5–24.8]	**0.0330**
	P_H2O_ (pt) (mbar)	6.4 [3.3–9.7]	6.9 [6.5–7.7]	**<0.0001**	6.7 [6.3–7.4]	9.0 [6.4–9.7]	**<0.0001**
	P_CO2_ (pt) (ppm)	241.5 [117.8–713.2]	450.5 [285.7–649.7]	**<0.0001**	319.3 [228.9–517.2]	682.0 [211.3–885.8]	**<0.0001**

HR, heart rate; RR, respiratory rate; HRmax %, percentage of maximum heart rate; Skin Temp, skin temperature; RMSSD, Root Mean square of successive RR interval differences; RR intervals, interbeat intervals between all successive heartbeats; SDNN, Standard Deviation of all NN intervals; NN intervals, interbeat intervals with artifacts removed; SDRR, standard deviation of RR intervals; CVSD, RMSSD divided by Mean NNI; pNNI-20, Percentage of successive RR intervals differing more than 20 ms; pNNI-50, Percentage of successive RR intervals that differing more than 50 ms; SDSD, SD of successive differences between NN; M-NNI, Mean of NN; CVNNI, SDNN divided by mean NN; LFnu, normalized Low-Frequency power; HFnu, normalized High-Frequency power; IA, indoor air; P_H2O_ (pt), water vapor partial pressure in the bedroom (patient’s contribution); P_CO2_ (pt), CO_2_ partial pressure in the bedroom (patient’s contribution); A.U., arbitrary units. Bold character indicates significant differences.

### 3.9 Effect of EEG alterations on continuous wearable sensor device and environmental variables

In addition, all the 24-h monitoring parameters were evaluated as a function of EEG findings of multifocal epileptiform activity and severe general background slowing (Table 5).

RTT patients with EEG multifocal epileptiform activity showed statistically increased HRmax % values (*p* = 0.0076) and reduced skin temperature values (*p* = 0.0120). No significant differences were observed for the HRV time-domain parameters except for CVNNI (*p* < 0.0001). Conversely, all the examined HRV frequency-domain variables were statistically different. In particular, we observed reduced values in LFnu (~38-fold, *p* < 0.0001) (*p* < 0.0001) and HFnu (*p* < 0.0001) together with increased LF/HF ratio (*p* < 0.0001) and total power values (approximately 2-fold, *p* < 0.0001) in RTT patients with EEG multifocal epileptiform features. Furthermore, for the IA monitoring, a decreased relative humidity (*p* < 0.0001) was found to be associated with a significant increase in both P_H2O_ (pt) (*p* < 0.0001) and P_CO2_ (pt) (approximately 1.9-fold, *p* < 0.0001) (Table 5).

The other key EEG abnormality (i.e., severe general background slowing) apparently shows a partial overlapping pattern with that observed in RTT patients with EEG multifocal epileptiform activity. For wearable sensor parameters, we found a statistically significant reduction in RR (approximately 1.3-fold, *p* = 0.012) and skin temperature values (*p* = 0.0279). For HRV time-domain parameters, the only statistically significant differences were observed for RMSSD values (−1.5-fold, *p* = 0.012), SDSD values (+1.5 fold, *p* = 0.012), and CVNNI values (+1.1, *p* = 0.027). Interestingly, for the HRV frequency-domain variables, HFnu values were increased (*p* < 0.0001), whereas LF/HF ratio (*p* < 0.0001) and total power levels (*p* < 0.0001) were reduced. Some IA variables [i.e., relative humidity, P_H2O_ (pt), and P_CO2_ (pt)] were overlapping with the pattern observed in patients with EEG multifocal epileptiform activity. Of note, P_H2O_ (pt) and P_CO2_ (pt) values were increased approximately 1.3-fold and 2.1-fold, respectively. Furthermore, increased CO_2_ levels (approximately 1.4-fold, *p* < 0.0001) and reduced temperature (*p* = 0.0330) were also evidenced for RTT patients with severe general background slowing (Table 5).

### 3.10 Effects of beta blockade on continuous wearable sensor device and environmental variables

An adolescent RTT patient was under treatment with the beta-blocker drug propranolol due to a prolonged QTc interval. Therefore, the potential influence of this drug on the 24-h biovital monitoring was examined. A significant decrease in HRmax % and skin temperature values was observed (*p* < 0.0001) (Supplementary Table S1). While no significant difference was observed in the HRV time-domain variables (*p* ≥ 0.1264), all the examined HRV frequency-domain parameters were statistically different from those of the remaining untreated RTT group. In particular, increased LFnu (approximately 847%, *p* < 0.0001), HFnu (*p* < 0.0001), and total power values (−14%, *p* = 0.0022) were observed, while the LF/HF ratio was decreased (approximately 36%, *p* < 0.0001). In the environment of the same RTT patient, significant increases in relative humidity (*p* < 0.0001), atmospheric pressure (*p* < 0.0001), and P_H2O_ (pt) values (*p* < 0.0001) were detectable as compared to the environmental variables measured in the untreated RTT patients (not on beta-blocker). Of note, a strong depressed HR/LF ratio in the patient was detectable (*p* < 0.0001).

### 3.11 Diurnal variations in minimum (nadir) and maximum (zenith) values for environmental variables and patient-derived parameters

To explore the possible role of sympathovagal imbalance on the daily activities/quality of life of the examined RTT patients, the frequency distribution of the daily minimum (nadir) and maximum (zenith) values for the measured environmental variables and the estimated patient-derived parameters P_H2O_ (pt) and P_CO2_ (pt) were evaluated (Supplementary Figure S4). A significant diurnal variation in the minimum values of RR (*p* = 0.039) and the maximum values of skin temperature were observed (*p* = 0.045) (Supplementary Figure S2A). For the patient-derived parameters, we observed statistically significant differences for the P_H2O_ (pt) and P_CO2_ (pt). In particular, the daily distribution of maximum values of P_H2O_ (pt) was statistically significant (*p* = 0.045), with a prevalence of 55% during the night, while the minimum value distribution of P_H2O_ (pt) was close to statistical significance (*p* = 0.051). Of note, for the diurnal distribution of P_CO2_ (pt), distinctively mirroring patterns in both minimum and maximum values were detectable (*p* = 0.020 and *p* = 0.001, respectively). Indeed, the minimum values of P_CO2_ (pt) were recorded during the morning time (approximately 90%), whereas the maximum values of P_CO2_ (pt) were found at night time (approximately 80%) (Supplementary Figure S4). Regarding the HRV time-domain variables, no statistical difference was evidenced for their daily distributions except for the maximum value distribution of M-NNI (*p* = 0.044) (Supplementary Figure S2B). Similarly, for the HRV frequency-domain parameters, no significant difference was found in their daily distributions, with the single exception for the maximum value daily variation of HFnu (a marker of PSNS activity), which maintained peak values during the night time (40%) (*p* = 0.005) (Supplementary Figure S4).

### 3.12 HR/LF ratio: relationships with age, key clinical RTT features, and disease severity scores

**Figure 5:**
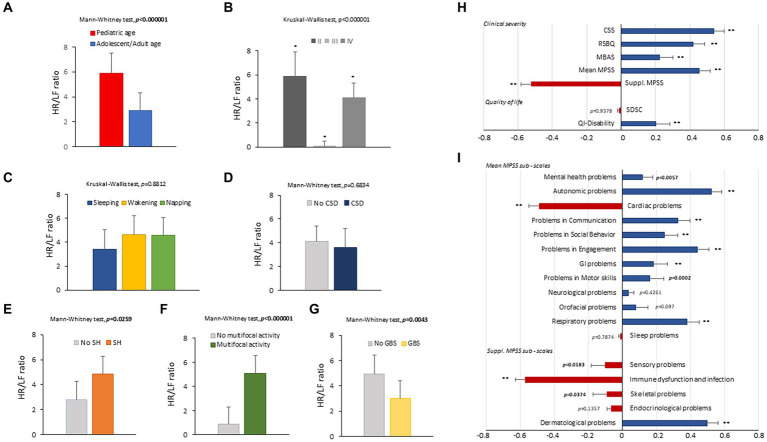
HR/LF ratio as a function of age **(A)**, disease stage **(B)**, sleep–wake cycle **(C)**, clinical sleep disorder **(D)**, subclinical hypoxia **(E)**, EEG finding of multifocal activity **(F)**, and EEG finding of general background slowing **(G)**. Spearman rank correlation coefficients (Rho) between the HR/LF ratio and clinical severity (CSS, RSBQ, MBAS, MPSS Main, MPSS supplement), quality of life, and sleep disorder **(H)**. Spearman rank correlation coefficients (Rho) between the HR/LF ratio and MPSS individual sub-items (**I**). HR, heart rate; LF, Low-Frequency power; CSD, clinical sleep disorders; SH, subclinical hypoxia; GBS, generalized background slowing; MPSS, Multi-System Profile of Symptoms Scale. Bold characters indicate significant differences; * *p* < 0.05, ** *p* < 0.0001, *** *p* < 0.000001.

HR/LF ratio was found to be increased in the pediatric patients as compared to the adolescent/adult group (*p* < 0.00001) (Figure 5A). This index was significantly reduced in stage III patients as compared with the other disease stages (*p* < 0.00001) (Figure 5B). No significant differences were detectable for the sleep–wake cycle (Figure 5C) and clinical sleep disorder (Figure 5D). An increased HR/LF ratio was evidenced in patients with subclinical hypoxia (*p* = 0.0259) (Figure 5E). EEG finding of multifocal activity was associated with significantly higher HR/LF ratio values (*p* < 0.00001) (Figure 5F). Conversely, reduced HR/LF ratios were associated with EEG findings of general background slowing (*p* = 0.0043) (Figure 5G). HR/LF was positively correlated with CSS, RSBQ, MBAS, MPSS Main, and QI Disability (Spearman rank correlation coefficient range: 0.204 to 0.540, *p* < 0.0001). Conversely, HR/LF was inversely related to supplement MPSS (Spearman rank correlation coefficient: −0.522, *p* < 0.0001). No significant association was found for SDSC (*p* = 0.9378) (Figure 5H). Furthermore, significant positive associations were observed between the HR/LF ratio and the subscores of MPSS mental health and autonomic, communication, social behavior, engagement, gastrointestinal, motor skills, and respiratory problems (Spearman rank correlation coefficient range: 0.118 to 0.522, *p* < 0.0001). Conversely, the HR/LF ratio was inversely related to the subscores of MPSS cardiac problems (Spearman rank correlation coefficient − 0.487, *p* < 0.0001).

The HR/LF ratio was positively related to the subscores of the MPSS dermatological problems (Spearman rank correlation coefficient: 0.499, *p* < 0.0001) while inversely associated with the subscore of MPSS sensory, immune dysfunction/infection, and skeletal problems (Spearman rank correlation coefficient range: −0.089 to −0.571, *p* ≤ 0.0374) (Figure 5I).

### 3.13 Clinical sleep disorder: ROC curve analysis

**Figure 6:**
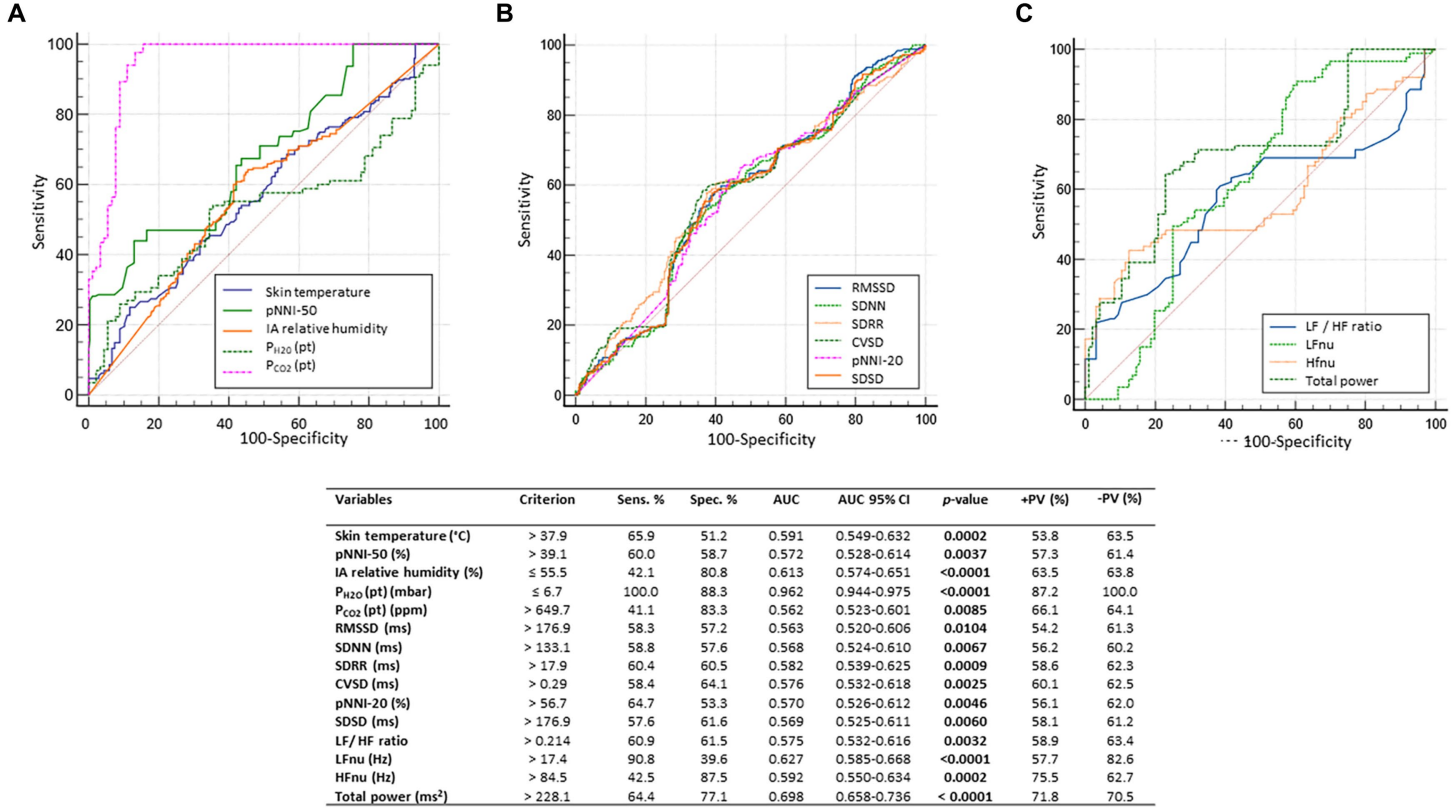
For the dependent variable clinical sleep disorder (CSD), the results of the receiver operating characteristic (ROC) curve analysis indicated a highly significant predictive value for P_H2O_ (pat): cutoff criterion ≤6.7 mbar; AUC 0.926, 95% CI: 0.944–0.975 (*p* < 0.0001), with very good discriminative performance (100% sensitivity, 88.3% specificity, 87.2% positive predictive value, and 100% negative predictive value). In addition, a series of variables (skin temperature, pNNI, IA relative humidity, P_H2O_ (pat), RMSSD, SDNN, SDRR, CVSD, pNNI-20, SDSD, LF/HF ratio, LFnu, HFnu, and total power) showed weak to fair discriminative performance (AUC range: 0.562 to 0.698; *p*-value range: <0.0001 to 0.0104). For the sake of clarity, predictor variables were grouped into 3 graphs (**A-C**). AUC, area under the curve; AUC, Area Under the Curve; SE, standard error; Sens, sensitivity; Spec, specificity; +LR, positive likelihood ratio; −LR, negative likelihood ratio; +PV, positive predictive value; −PV, negative predictive value; Skin Temp, skin temperature; RMSSD, Root Mean square of successive RR interval differences; RR intervals, interbeat intervals between all successive heartbeats; SDNN, Standard Deviation of all NN intervals; NN intervals, interbeat intervals from which artifacts have been removed; CVSD, RMSSD divided MeanNNI; pNNI-20, Percentage of successive R-R intervals that differ by more than 20 ms; pNNI-50, Percentage of successive R-R intervals that differ by more than 50 ms; SDSD, SD of successive differences between NN; M-NNI, Mean of NN. CVNNI, SDNN divided by mean NN; IA, indoor air; P_H2O_ (pt), water vapor partial pressure originating from patients in the bedroom; P_CO2_ (pt), CO_2_ partial pressure originating from patients in the bedroom; LFnu, normalized Low-Frequency power; HFnu, normalized High-Frequency power. Bold characters indicate significant differences.

From the ROC curve analysis, a value of P_H2O_ (pat) ≤6.7 mbar was found to be a significant predictor for a clinical sleep disorder, with very good discriminative performance (AUC 0.962, 95% CI: 0.944–0.975 (*p* < 0.0001), 100% sensitivity, 88.3% specificity, 87.2% positive predictive values, and 100% negative predictive values). In addition, weak to fair discriminative performances (AUC range: 0.562 to 0.698; *p*-value range: <0.0001 to 0.0104) were evidenced for a number of biovitals and HRV time-domain and frequency-domain parameters, together with environmental variables (skin temperature, pNNI-20, IA relative humidity, P_CO2_ (pt), RMSSD, SDNN, SDRR, CVSD, pNNI, SDSD, LF/HF ratio, LFnu, HFnu, and total power) (Figure 6).

### 3.14 Subclinical hypoxia: ROC curve analysis

**Figure 7:**
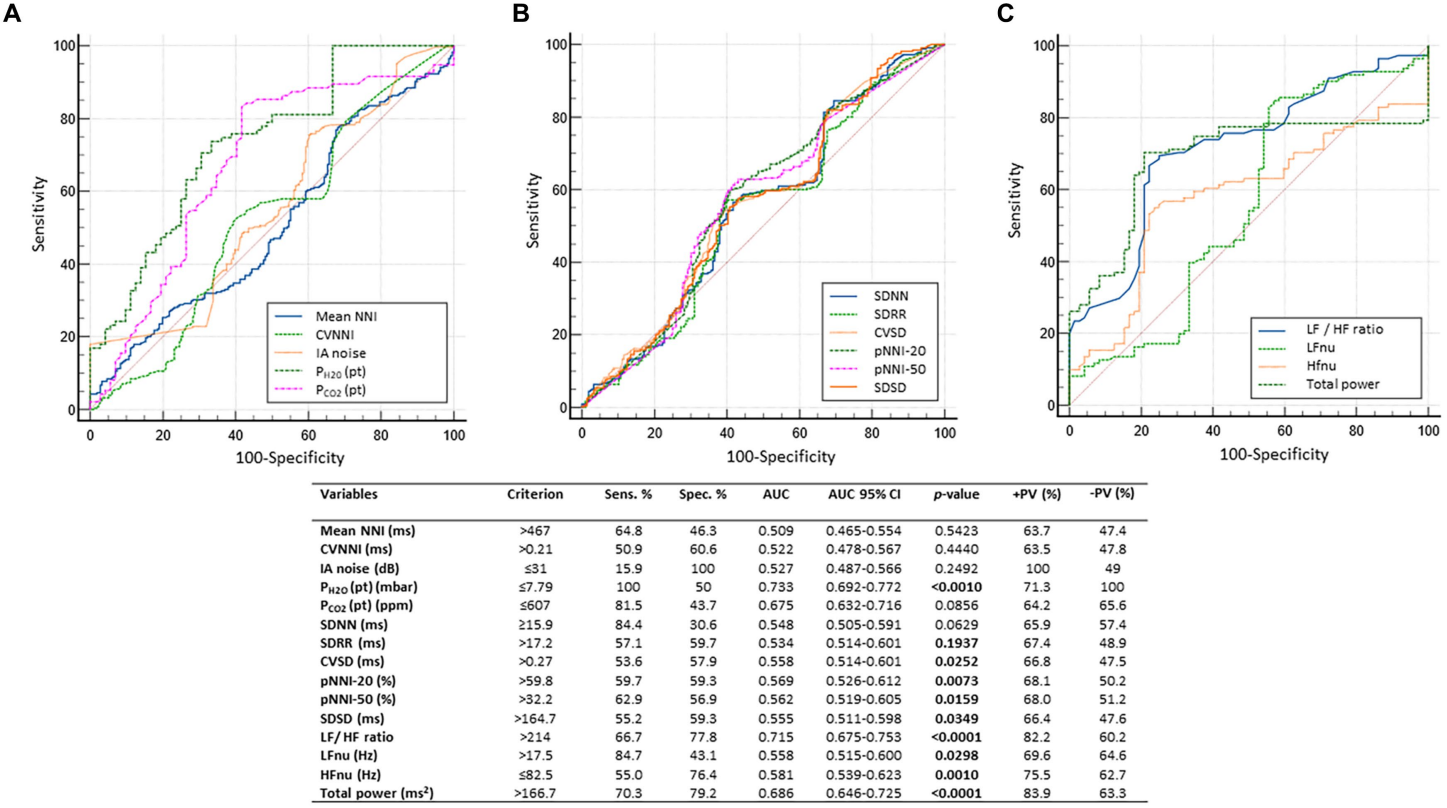
Dependent variable of subclinical hypoxia. Receiver operating characteristic (ROC) curve analysis indicated a series of statistically significant predictive variables with either acceptable [variables: P_H2O_ (pat), LF/HF ratio, and total spectral power; AUC range 0.686 to 0.733, *p*-value range: <0.0001 to 0.0010] or weak discriminative performance (variables: CVSD, pNNI-20, pNNI-50, SDSD, LFnu, and HFnu; AUC range: 0.534 to 0.581; *p*-value range: 0.001 to 0.0349). For the sake of clarity, predictor variables were grouped into 3 graphs (**A-C**) AUC, area under the curve; AUC, Area Under the Curve; SE, standard error; Sens., sensitivity; Spec, specificity; +LR, positive likelihood ratio; −LR, negative likelihood ratio; +PV, positive predictive value; −PV, negative predictive value; R-R intervals, interbeat intervals between all successive heartbeats; SDNN, Standard Deviation of all NN intervals; NN intervals, interbeat intervals from which artifacts have been removed; CVSD, RMSSD/MeanNNI ratio; pNNI-20, Percentage of successive R-R intervals that differ by more than 20 ms; pNNI-50, Percentage of successive R-R intervals that differ by more than 50 ms; P_H2O_ (pt), water vapor partial pressure originating from patients in the bedroom; P_CO2_ (pt), CO_2_ partial pressure originating from patients in the bedroom. Bold characters indicate significant differences.

The results of the ROC curve analysis indicated a series of significant predictive variables for the dependent variable of subclinical hypoxia showing acceptable (variables: P_H2O_ (pt), LF/HF ratio, and total power; AUC range 0.686 to 0.733, *p*-value range: <0.0001 to 0.0010)or weak discriminative performances (variables: CVSD, pNNI-20, pNNI-50, SDSD, LFnu, and HFnu; AUC range: 0.534 to 0.581; *p*-value range: 0.001 to 0.0349) (Figure 7).

### 3.15 Clinical sleep disorder, subclinical hypoxia, and EEG alterations: stepwise multiple regression models

**Figure 8:**
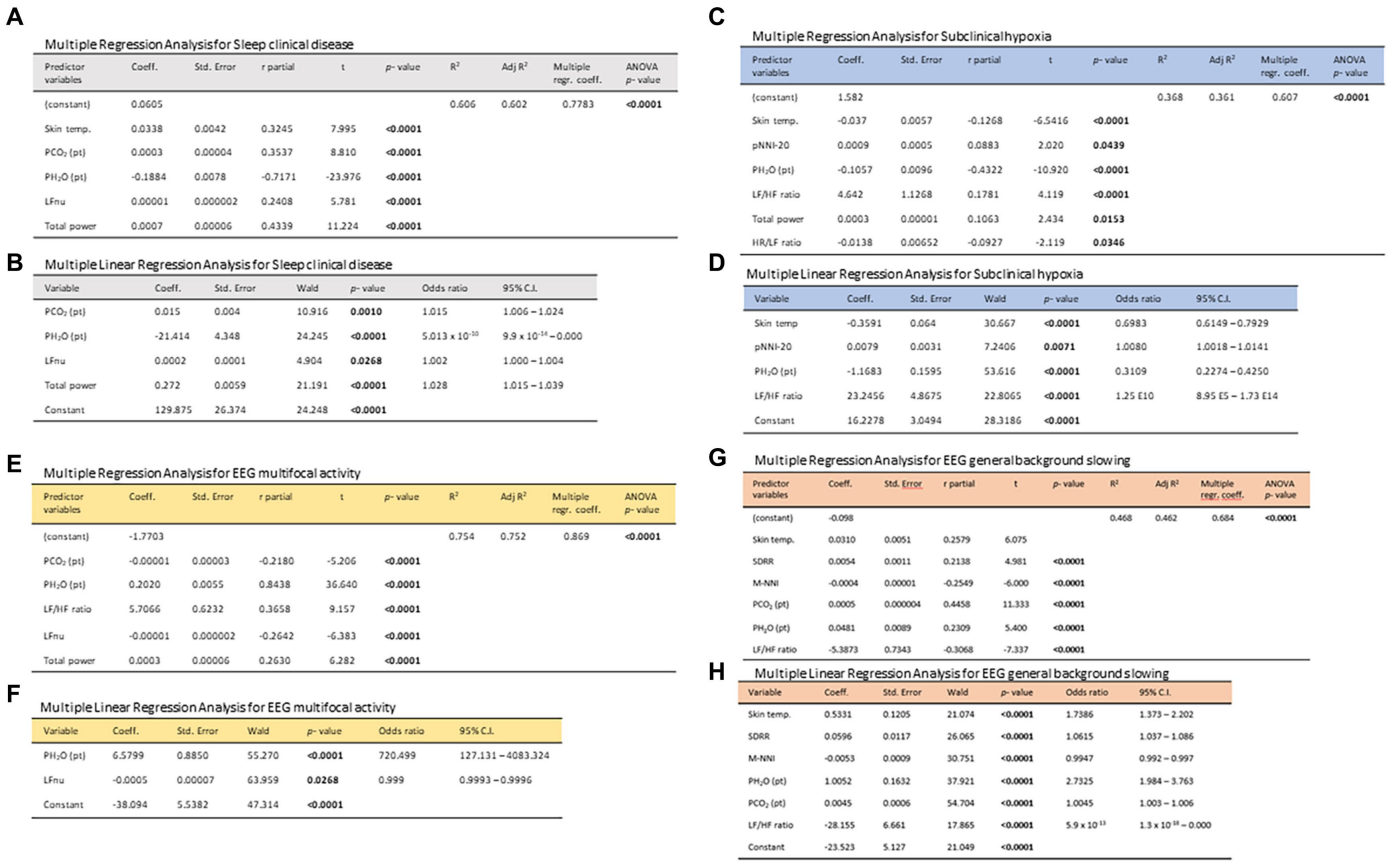
Stepwise multiple regression models and multivariate logistic regression models in the exploration of the relationships between 24 h-monitored biovitals/patient-derived environmental parameters as potential predictor variables and the dependent variables clinical sleep disorder **(A,B)**, subclinical hypoxia **(C,D)** EEG finding of multifocal activity **(E,F)**, and EEG finding of general background slowing **(G,H)**.

From a multiple stepwise regression analysis model, clinical sleep disorder was found to be positively correlated with skin temperature, P_CO2_ (Pt), LFnu, and total power while inversely correlated with P_H2O_ (Pt); R^2^ 0.606, adjusted R^2^ 0.602, multiple regression coefficient 0.7783, *p* < 0.0001 (Figure 8A). In a multivariate stepwise logistic regression analysis, clinical sleep disease in RTT patients was positively associated with P_CO2_ (Pt) (OR: 1.015, *p* = 0.001), LFnu (OR: 1.002, *p* = 0.0268), and total power (OR: 1.028, *p* < 0.0001) while inversely correlated with P_H2O_ (Pt) (OR: 5.013 ∙10^−10^, *p* < 0.0001) (Figure 8B). Multiple stepwise regression analysis model of subclinical hypoxia in RTT patients was positively correlated with p-NNI-20, LF/HF ratio, and total power while inversely related to skin temperature, P_H2O_ (Pt), and HR/LF ratio; R^2^ 0.368, adjusted R^2^ 0.361, multiple regression coefficient 0.607, *p* < 0.0001 (ANOVA) (Figure 8C). Multivariate stepwise logistic regression analysis of subclinical hypoxia in RTT patients was found to be positively related to p-NNI-20 (OR: 1.008, *p* = 0.0071) and LF/HF ratio (OR: 1.25 E10, *p* < 0.0001) while inversely related to skin temperature (OR: 0.6983, *p* < 0.0001) and P_H2O_ (Pt) (OR: 0.3109, *p* < 0.0001) (Figure 8D). Moreover, a multiple stepwise regression analysis model of EEG multifocal activity in RTT patients was found to be positively correlated with P_H2O_ (Pt), LF/HF ratio, and total power while inversely correlated with P_CO2_ (Pt) and LFnu; R^2^ 0.754, adjusted R^2^ 0.752, multiple regression coefficient 0.869, *p* < 0.0001 (ANOVA) (Figure 8E). A multivariate stepwise logistic regression analysis, EEG multifocal activity in RTT patients, was positively correlated with p-NNI-20 (OR: 1.008, *p* = 0.0112), P_CO2_ (Pt) (OR: 1.015, *p* = 0.001), LFnu (OR: 1.002, *p* = 0.0268), and total power (OR: 1.0014, *p* = 0.0214) while inversely correlated with skin temperature (OR: 0.6983, *p* < 0.0001), LFnu (OR: 0.999, *p* < 0.0001), and P_H2O_ (Pt) (OR: 0.2779, *p* < 0.0001) (Figure 8F). Furthermore, a multiple stepwise regression analysis model, EEG general background slowing in RTT patients, was positively correlated with skin temperature, SDRR, P_CO2_ (Pt), and P_H2O_ (Pt), while inversely correlated with Mean NNI and LF/HF ratio; R^2^ 0.468, adjusted R^2^ 0.462, multiple regression coefficient 0.684, *p* < 0.0001 (ANOVA) (Figure 8G). A multivariate stepwise logistic regression analysis of EEG general background slowing in RTT patients was found to be positively correlated with skin temperature (OR: 1.7386, *p* < 0.0001), SDRR (OR: 1.0615, *p* < 0.0001), P_H2O_ (Pt) (OR: 2.7325, *p* < 0.0001), P_CO2_ (Pt) (OR: 1.0045, *p* < 0.0001), and LF/HF ratio (OR: 5.9 10^13^, *p* < 0.0001), while inversely correlated with Mean NNI (OR: 0.9947, *p* < 0.0001) (Figure 8H).

## 4 Discussion

RTT is known to have an increased mortality risk, previously linked to cardiorespiratory issues (24, 76) or pneumonia (77). Despite the changing survival rates, due to carefully addressing modifiable risk factors (76), overall clinical severity remains a key player in the mortality risk in classic RTT (76, 77). To date, four prior studies using continuous monitoring of vital signs by different wearable sensor devices have explored variations of biovital/physiological signs and HRV metrics in RTT (19, 45–47). The present pilot study demonstrates the feasibility of a continuous 24-h in-home non-invasive biovital signs monitoring by a wearable sensor device, coupled with continuous in-home monitoring of environmental indoor variables in classic RTT patients. The percentage of correctly measured and recorded data in the absence of significant adverse events was appreciable (>70%). Apart from swimming, this innovative wearable sensor technology allowed the patients to continue their life under normal conditions and activities without restrictions.

Our results are only partially in line with those of prior investigations (45, 46) regarding age, sleep–wake cycle, *MECP2* gene mutation category, and clinical severity.

Resting sinus tachycardia and a reduced HRV in RTT patients are currently thought to be the result of ANSD (3, 19, 20, 78). In particular, HRV has been exploited in a novel integrated autonomic-sensory measurement approach for investigating somatosensory function (79)where an abnormal HRV could improve risk stratification (80). Efforts were necessary to improve the understanding of heart–brain interactions (81). Influences between the heart and the brain include autonomic regulation and hemodynamic connections and HRV acting as a proxy of autonomic activity, which is associated with executive functions, decision-making, and emotional regulation (81). While a high HRV is indicative of adaptability and good health, a reduced HRV variability (i.e., a decreased vagal functioning) is reported to be a sign of inadequate adaptability and physiological dysfunction (82, 83). While technological advancements have made it possible to acquire high-quality HR and HRV data in resting conditions, several questions remain unanswered regarding the use of resting heart rate and HRV in a population and inter-individual (i.e., in response to stressors) levels (84). Previous research has characterized HRV (16, 20–23, 85), cardiac repolarization (28, 29), and cardiorespiratory coupling to be valuable measures of autonomic dysregulation (21, 23, 30–32). Dysregulation of autonomic circuits represents a key feature of RTT, possibly contributing to sudden death (21, 24, 25, 27, 28, 78, 86, 87). Our study shows that HRV metrics (both time-domain and frequency-domain parameters) are related to age (in that pediatric patients show higher values than adolescent/adult patients with the exception of LFnu), clinical stage, and *MECP2* gene mutations category. Sleep–wake cycle impacted the HRV frequency-domain with a distinct pattern as a function of age. Clinical sleep disorder (CSD) was found to be related to higher values for five out of nine HRV time-domain parameters in pediatric patients, whereas no differences were observed in the adolescent/adult group. HRV frequency-domain parameters were all increased in pediatric patients with clinical sleep disorders. The pattern of the LF/HF ratio is reduced in adolescent/adult patients with clinical sleep disorders faced with increased spectral total power. The results of the present study indicate that the HRV metrics *per se* (i.e., the commonly used time-domain and frequency-domain indices) are unable to explain illness severity and several other features of the RTT phenotype. Overall, in the present study, highly reduced HRV values (RMSSD<20 ms) were observed in less than one-quarter of the analyzable records (data not shown). Therefore, our results would not confirm the findings of a generally reduced HRV in RTT patients as reported in at least two previous monitoring studies using wearable sensor devices (45, 46). Several factors, including methodology differences, may likely account for this apparent discrepancy. In the present study, we can infer a persistent fatigue status, with a significant autonomic tone shift toward SNS predominance over the PSNS.

To date, the pathophysiological role of *MECP2* gene mutations in the etiology of intrinsic cardiac abnormality and sudden death remains unclear. Several lines of evidence suggest the co-existence of intrinsic structural myocardial abnormalities in RTT patients (88). In particular, it has been recently reported that cardiac repolarization abnormalities are present in RTT patients, even without long QTc, and the T-wave morphology is related to the RTT genotype, which is a predictor of mortality (89). A subclinical myocardial dysfunction in RTT patients has been previously reported, where a mild-to-moderate decrease in systolic and diastolic left and right ventricles longitudinal function in both typical and atypical RTT without evidence of QT prolongation was found (26). MeCP2, a reader of DNA methylation and a component of a co-repressor complex, has been shown to regulate gene expression in chronic heart failure (90) and to attenuate *in vitro* hypoxia/reperfusion-induced injury in H9c2 cardiomyocytes by modulating the SFRP4/Wnt/β-catenin axis (91). Moreover, an altered Wnt signaling, consequent to MeCP2 protein deficiency, is associated with an abnormal cardiac ion channel expression and cellular electrophysiology underlying QT prolongation and sudden death in RTT (86).

Severe hypotonia is a recognized feature in RTT patients and MeCP2-deficient mice. Specifically, mild generalized hypotonia is frequently observed in the first months of life in RTT patients, with an abnormal muscle tone generally observed later (6). Exercise fatigue in RTT has been reported in both patients (33) and experimental models recapitulating the disease (34). It is postulated that the RTT phenotype is due to central and peripheral MeCP2 protein deficiency. Indeed, a comparison of peripheral knock-out (PKO) mice with wild-type and global Mecp2-deficient mice (36) showed that the majority of RTT-associated behavioral, sensorimotor, gait, and autonomic (respiratory and cardiac) phenotypes are dependent on CNS deficiency; most notably, hypo-activity, exercise fatigue, and bone abnormalities have been reported to depend on peripheral Mecp2 deficiency. In particular, a disorganized architecture with hypotrophic fibers and tissue fibrosis in the skeletal muscles and altered IGF-1/Akt/mTOR pathway have been demonstrated in *Mecp2*-null mice (92). These data suggest that hypotonia is mainly, if not exclusively, mediated by non-cell autonomous effects and support the hypothesis that defects in the paracrine/endocrine signaling system (in particular, GH/IGF axis) are the main cause of the observed muscular defects in RTT (92).

In the current study, besides exploiting biovital measurements and the standard HRV metrics, HR max % (maximal heart rate) and the HR/LF ratio were evaluated as potential markers of persistent fatigue and SNS activation under dynamic exercise, respectively. HR max % is an essential measure of cardiovascular compliance to exercise testing and exertion during exercise despite the commonly used equations for evaluating age-predicted HR max %, which showed poor agreement with the actual value measured by graded treadmill exercise tests in cooperating subjects (93). Nonetheless, the testing of dynamical exercise in RTT patients using traditional methods is challenging, while continuous monitoring of HR and HRV metrics during a sufficiently prolonged time period (i.e., the present study) offers an alternative way to identify individually based HR max %. The present research suggests that the HR max % of RTT patients is persistently in the target ranges comparable with those observed during vigorous exercise.

A persistent fatigue status, with significant autonomic tone shift toward SNS predominance over the PSNS, could be inferred from our continuous non-invasive 24-h in-home monitoring in RTT patients. In the present study, for the first time, two markers of fatigue, i.e., HR Max % (63) and HR/LF (67), were applied to the interpretation of the continuous biovital monitoring in our particular patient group. A comparison of RTT patients with healthy young subjects under intense exercise evidenced the relevance of the HR/LF ratio for gauging non-invasively the clinical disease severity of the disease. When the HRV-derived HR/LF ratio, previously confined to sports physiology and medicine (67), was applied to the interpretation of the biovital monitoring of our examined RTT patients, it was found that several of the features of the RTT phenotype were relatable, strongly suggesting that a new point-of-view is warranted. Exercise-induced lactic threshold is the exercise intensity at which blood concentration of lactate begins to increase rapidly and is often expressed as 85% of HR max % or 75% of maximum oxygen intake (94).

Although exercise-induced lactic threshold has not been determined experimentally in RTT patients, evidence of systemic oxidative stress is well-established (95), as well as in experimental mouse models recapitulating the disease (96, 97). In the present study, the HR/LF ratio showed a significant relationship with subclinical hypoxia and key EEG. Epileptic seizures affect both sympathetic and parasympathetic nervous systems, thus leading to changes in the cardiac autonomic nervous function (98). In addition, HRV changes in the preictal phase have been previously reported (99). Among the time-domain parameters, patients with EEG multifocal epileptiform activity showed increased CVNNI, LF/HF, and total power values. Conversely, a different pattern for HRV time-domain parameters was observed in patients with EEG findings of background slowing with depressed HRV frequency-domain parameters. In our study, several parameters were found to be related to EEG findings of multifocal epileptiform activity and/or EEG background slowing, although the reasons behind the observed associations are yet to be elucidated.

In the present study, patients’ contributions to the environmental water vapor [P_H2O_ (pt)] and carbon dioxide [P_CO2_ (pt)] partial pressures were estimated and derived from the data collected by the meteorological station. Moreover, the (heat) energy lost by the patients to maintain body temperature and the skin–environment temperature difference (measured by the smart t-shirt sensor) were calculated. Although at a very early stage, correlating these data with the environmental and some biophysical parameters brought some interesting results. Regardless, given their potential relevance, these preliminary outcomes need confirmation by future independent studies. Coherently with the limits of the present pilot investigation and considering that no definitive conclusion can be drawn at this point, here we attempted to outline a possible rationale by theoretical interpretation.

The mechanism leading to heat loss and water evaporation can be explained by considering Eq. 1 and a realistic temperature difference between the environment and the body (e.g.: $T_{env}=15$ °C and $T_{body}=36$ °C). For instance, an atmospheric $RH_{env}$ of 70%, means that the air inhaled by a subject contains a water vapor partial pressure approximately $P_{H2O}\left(env\right)=11.7$ mbar [the saturation water vapor pressure of $P_{H2O}^{0}\left(T_{env}\right)\;\mathrm{is approximately}\;16.7$ mbar, as dictated by Eq. 1]. When the gas from the air reaches the body’s airways, it is naturally heated by the body (temperature of $T_{body}=36.0^{{}^{\circ}}C$ ), a temperature at which the saturation water vapor pressure, $P_{H2O}^{0}\left(T_{body}\right),\mathrm{increases}\;\mathrm{by}\;\mathrm{approximately}\;to\;56.7$ mbar. Hence, according to Eq. 1, the relative humidity, $RH_{body}$ , of the inhaled air suddenly drops to 20.6% , as long as the water vapor partial pressure (i.e., water content) is constant. Such a low $RH_{body}\;of\;20.6\%$ represents a typical humidity of desertic regions and triggers the mechanism of water evaporation from airway tissues. Water evaporation implies heating up to 100 °C and the compensation of the latent heat of evaporation [approximately 539 Kcal/kg (2,257 kJ/kg)], resulting in heat loss from body tissues. Hence, the body–environment temperature difference drives the water evaporation from the patient’s body (her contribution to environmental humidity) and the associated heat loss. On the other hand, the patient emission of CO_2_ certainly is related to oxygen consumption, which, in turn, is driven by physical activity (i.e., production of mechanical energy) and thermoregulation. Considering the facts above, identifying a relationship between the environmental and biophysical parameters is logical. Of course, further studies are needed to validate these new preliminary variables and to better investigate the environment–patient axis interactions and the potential use of them as new disease biomarkers.

Previous research indicates that the lungs can be primarily involved in the pathophysiology of respiratory dysfunction in RTT. A possible mechanistic explanation comes from the observation of a surfactant deficiency in Mecp2-deficient mice as a consequence of a previously unrecognized lung lipid perturbation due to accumulation of neutral lipids in the lung against decreased surfactant phospholipids (100). potentially contributing Intriguingly, surfactant dysfunction causes ground glass opacities and bronchial thickening, which is comparable with the computed tomography observation in RTT patient lungs (14). However, the role of changes in P_H2O_ (pt) in the RTT severity and progression should be considered in the study and certainly worthwhile for further investigation.

It is known that water evaporation is mainly driven by temperature differences and the difference between body and environment temperature could play a central role in the explanation of our findings. Of course, further studies are needed to validate these new preliminary variables and better investigate the environment–patient interactions and the potential use of them as new disease biomarkers.

Continuous monitoring of vital signs has potential value in the diagnosis and/or rescue of critically ill patients (101). In parallel with the technology of wearable wireless sensors for vital signs monitoring, the use of quantitative data and mathematical models to understand and treat disease is advancing rapidly.

Future studies are needed to address the possible role of the identified environmental variables and deepen the present investigation by complementing the results with a multicentric case–control design, complimentary polysomnography and kinetic analyses, and interfacing the biochemical correlates of the 24-h continuous non-invasive multiparameter home monitoring in RTT patients.

## Data availability statement

The raw data supporting the conclusions of this article will be made available by the authors, without undue reservation.

## Ethics statement

The studies involving humans were approved by Ethical Committee Regione Toscana – Area Vasta Sud Est. The study was conducted in accordance with the local legislation and institutional requirements. Written informed consent for participation in this study was provided by the participants' legal guardians/next of kin.

## Author contributions

SL: Conceptualization, Data curation, Formal analysis, Funding acquisition, Methodology, Project administration, Visualization, Writing – original draft, Writing – review & editing. LB: Data curation, Funding acquisition, Project administration, Visualization, Writing – review & editing. SDL: Data curation, Investigation, Methodology, Writing – review & editing. AmB: Methodology, Writing – review & editing, Supervision. VS: Writing – review & editing, Investigation. AV: Writing – review & editing, Methodology, Supervision. MCP: Methodology, Supervision, Writing – review & editing. GP: Investigation, Methodology, Writing – review & editing. LN: Methodology, Supervision, Writing – review & editing. AGN: Investigation, Writing – review & editing. GDR: Methodology, Supervision, Writing – review & editing. MBTC: Investigation, Methodology, Writing – review & editing. RC: Methodology, Supervision, Writing – review & editing. SG: Methodology, Supervision, Writing – review & editing. GL: Formal analysis, Supervision, Writing – review & editing. ET: Formal analysis, Methodology, Supervision, Writing – original draft, Writing – review & editing. PM: Methodology, Supervision, Writing – review & editing. MB: Formal analysis, Methodology, Software, Supervision, Validation, Writing – review & editing. AlB: Conceptualization, Data curation, Formal analysis, Software, Validation, Visualization, Writing – original draft, Writing – review & editing. CDF: Conceptualization, Data curation, Formal analysis, Funding acquisition, Investigation, Methodology, Project administration, Resources, Visualization, Writing – original draft, Writing – review & editing.

## Glossary

ANSD: Autonomic nervous system dysfunction
ASM: anti-seizure medication
A.U.: arbitrary units
AUC: Area Under the Curve
CO_2_: carbon dioxide
CSD: Clinical Sleep Disorder
CSS: Rett Clinical Severity Score
CVNNI: SDNN divided by mean N-N
CVSD: RMSSD to Mean NNI ratio
ECG: electrocardiography
EEG: electroencephalography
EIA: exercise-induced asthma
HF: High-frequency power
HFnu: normalized High-Frequency power
HR: Heart Rate
HRmax %: percentage of maximum HR
HRV: Heart Rate Variability
IAQ: Indoor Air Quality
LF: Low-frequency power
LFnu: normalized Low-Frequency power
LF/HF: Low-frequency power to high-frequency power ratio
MBAS: Motor Behavioral Assessment Scale
*MECP2*: Methyl-CpG-binding protein 2 gene
M-NNI: mean of N-N
MPSS: Multi-System Profile of Symptoms Scale
N-N intervals: interbeat intervals from which artifacts have been removed
P_CO2_ (pt): the patient contribution to the CO_2_ partial pressure
P_H2O_ (pt): the patient contribution to the H_2_O partial pressure
pNNI-20: percentage of successive R-R intervals that differ by more than 20 ms
pNNI-50: percentage of successive R-R intervals that differ by more than 50 ms
QI-Disability: Quality-of-Life Inventory-Disability
RSBQ: Rett Syndrome Behavior Questionnaire
CSD: Rett Clinical Severity Score
RMSSD: Root Mean square of successive R-R interval differences
RR: Respiratory Rate
R-R intervals: interbeat intervals between all successive heartbeats
RTT: Rett syndrome
SD: standard deviation
SDNN: SD of all N-N intervals
SDRR: SD of R-R intervals
SDSC: Sleep Disturbance Scale for Children Questionnaire
SDSD: SD of successive differences between N-N
SkT: skin temperature
