# Supplementary material for: 24-h continuous non-invasive multiparameter home monitoring of vitals in patients with Rett syndrome by an innovative wearable technology: evidence of an overlooked chronic fatigue status
Source: Front Neurol. 2024 Jun 17;15:1388506. doi: 10.3389/fneur.2024.1388506 (PMC11215834; doi:10.3389/fneur.2024.1388506)
Supplement: Supplementary file 1 [file Data_Sheet_1.DOCX]

Supplementary Material

24-hour continuous noninvasive multiparameter home monitoring of vitals in patients with Rett syndrome by an innovative wearable technology: Evidence of an overlooked chronic fatigue status

**Silvia Leoncini*, Lidia Boasiako, Sofia Di Lucia, Amir Beker, Valeria Scandurra, Aglaia Vignoli, Maria Paola Canevini, Giulia Prato, Lino Nobili, Antonio Gennaro Nicotera, Gabriella Di Rosa, Maria Beatrice Testa Chiarini, Renato Cutrera, Salvatore Grosso, Giacomo Lazzeri, Enrico Tongiorgi, Pasquale Morano, Matteo Botteghi, Alessandro Barducci, Claudio De Felice***

*** Correspondence:** Corresponding Authors: [geniente@gmail.com](mailto:geniente@gmail.com) and [s.leoncini74@gmail.com](mailto:s.leoncini74@gmail.com)


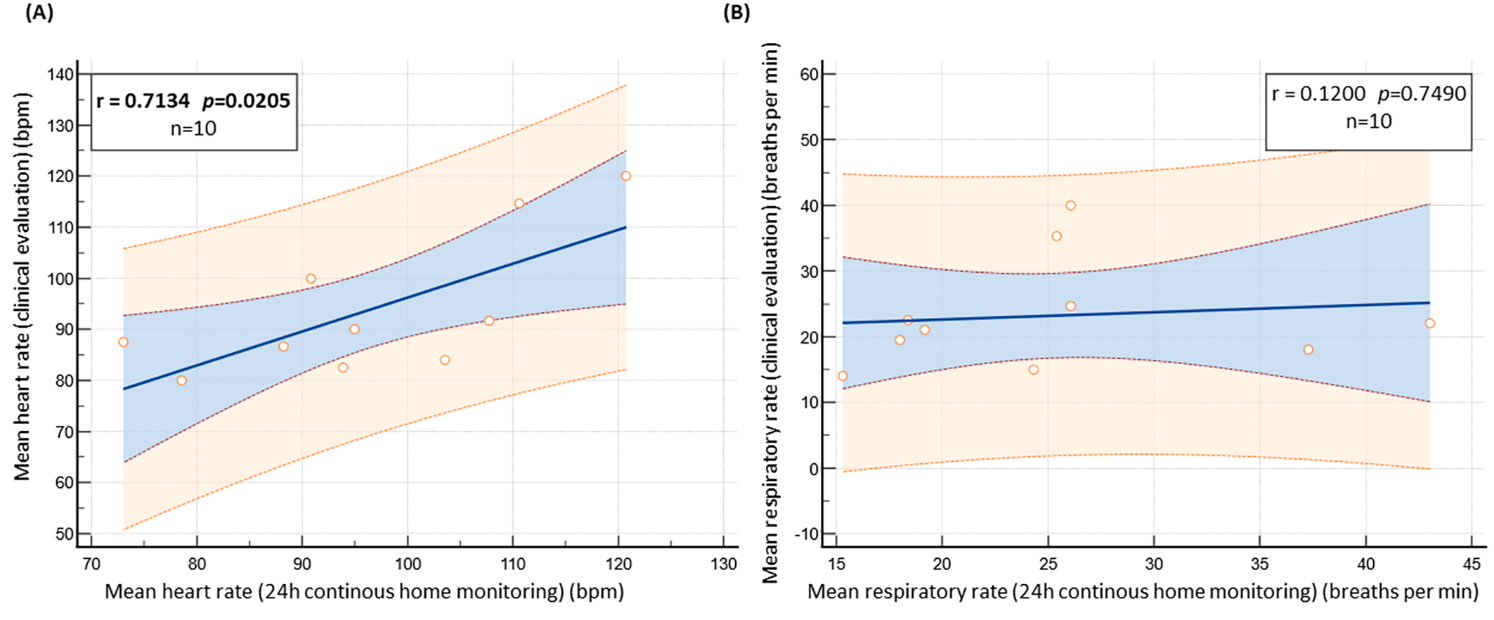


**Supplementary Figure 1.** Significant correlation between heart rate as measured in the clinical setting and the same biovital parameter as measured by 24-hours continuous noninvasive multiparameter home monitoring (A). No statistically significant relationship was observed between respiratory rate measured in the clinical setting and the results of the same biovital parameter as measured by 24-hour continuous noninvasive multiparameter home monitoring (B). Bold characters indicate statistically significant differences.
