# Supplementary material for: 24-h continuous non-invasive multiparameter home monitoring of vitals in patients with Rett syndrome by an innovative wearable technology: evidence of an overlooked chronic fatigue status
Source: Front Neurol. 2024 Jun 17;15:1388506. doi: 10.3389/fneur.2024.1388506 (PMC11215834; doi:10.3389/fneur.2024.1388506)
Supplement: Supplementary file 2 [file Data_Sheet_2.DOCX]

Supplementary Material

24-hour continuous noninvasive multiparameter home monitoring of vitals in patients with Rett syndrome by an innovative wearable technology: Evidence of an overlooked chronic fatigue status

**Silvia Leoncini*, Lidia Boasiako, Sofia Di Lucia, Amir Beker, Valeria Scandurra, Aglaia Vignoli, Maria Paola Canevini, Giulia Prato, Lino Nobili, Antonio Gennaro Nicotera, Gabriella Di Rosa, Maria Beatrice Testa Chiarini, Renato Cutrera, Salvatore Grosso, Giacomo Lazzeri, Enrico Tongiorgi, Pasquale Morano, Matteo Botteghi, Alessandro Barducci, Claudio De Felice***

*** Correspondence:** Corresponding Authors: [geniente@gmail.com](mailto:geniente@gmail.com) and [s.leoncini74@gmail.com](mailto:s.leoncini74@gmail.com)


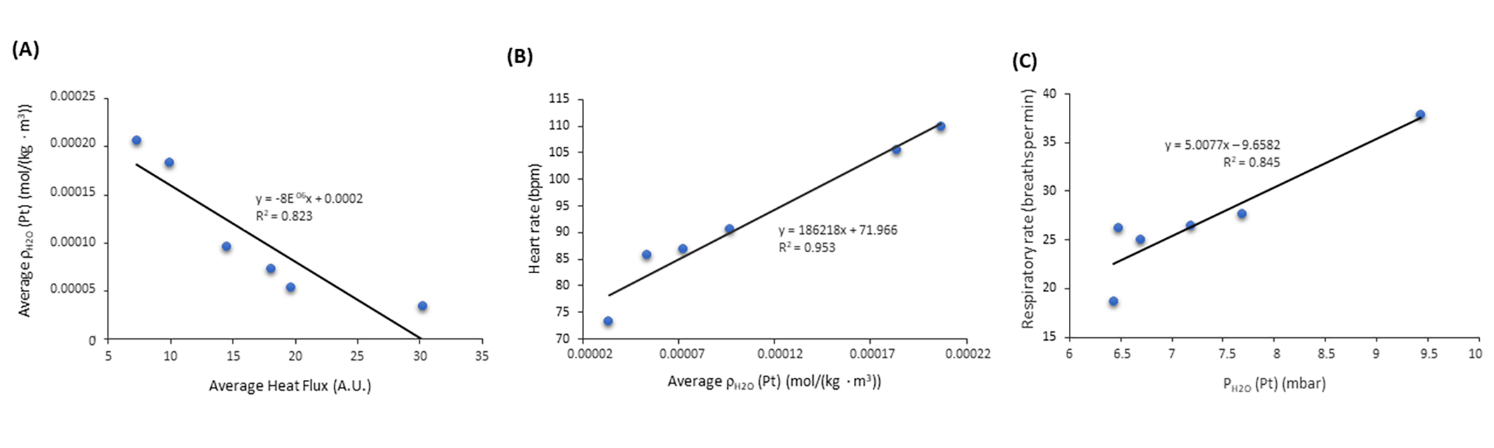


**Supplementary Figure 2. (A)**Significant inverse correlation between ρ_H2O_(pt) and the estimated flux of heat lost by the patient due to the patient-environment temperature difference. The heat flux has been estimated by means of skin temperature data collected by the wearable t-shirt, the environment temperature acquired by the meteorological station, and patient’s height and mass. **(B)**Significant positive correlation between heart rate and ρ_H2O (pt)_.**(C)**Positive correlation between RR and P_H2O_ (pt), indicating the potential role of increased RR in contributing to of the emitted water vapor. The patient’s contribution to the water vapor molar density (per body mass unit) has been derived and from the relative humidity measured by the weather station with indoor sensors. The data showed in these scatterplots also are an indirect partial validation of the physical/mathematical modelling adopted for estimating patient parameters from the collected environmental data.

Abbreviations: ρ_H2O_(pt): water vapor relative molar density (per body mass unit).  P_H2O_ (pt): water vapor partial pressure originating from patients in the bedroom.
