# Supplementary material for: 24-h continuous non-invasive multiparameter home monitoring of vitals in patients with Rett syndrome by an innovative wearable technology: evidence of an overlooked chronic fatigue status
Source: Front Neurol. 2024 Jun 17;15:1388506. doi: 10.3389/fneur.2024.1388506 (PMC11215834; doi:10.3389/fneur.2024.1388506)
Supplement: Supplementary file 3 [file Data_Sheet_3.DOCX]

Supplementary Material

24-hour continuous noninvasive multiparameter home monitoring of vitals in patients with Rett syndrome by an innovative wearable technology: Evidence of an overlooked chronic fatigue status

**Silvia Leoncini*, Lidia Boasiako, Sofia Di Lucia, Amir Beker, Valeria Scandurra, Aglaia Vignoli, Maria Paola Canevini, Giulia Prato, Lino Nobili, Antonio Gennaro Nicotera, Gabriella Di Rosa, Maria Beatrice Testa Chiarini, Renato Cutrera, Salvatore Grosso, Giacomo Lazzeri, Enrico Tongiorgi, Pasquale Morano, Matteo Botteghi, Alessandro Barducci, Claudio De Felice***

*** Correspondence:** Corresponding Authors: [geniente@gmail.com](mailto:geniente@gmail.com) and [s.leoncini74@gmail.com](mailto:s.leoncini74@gmail.com)


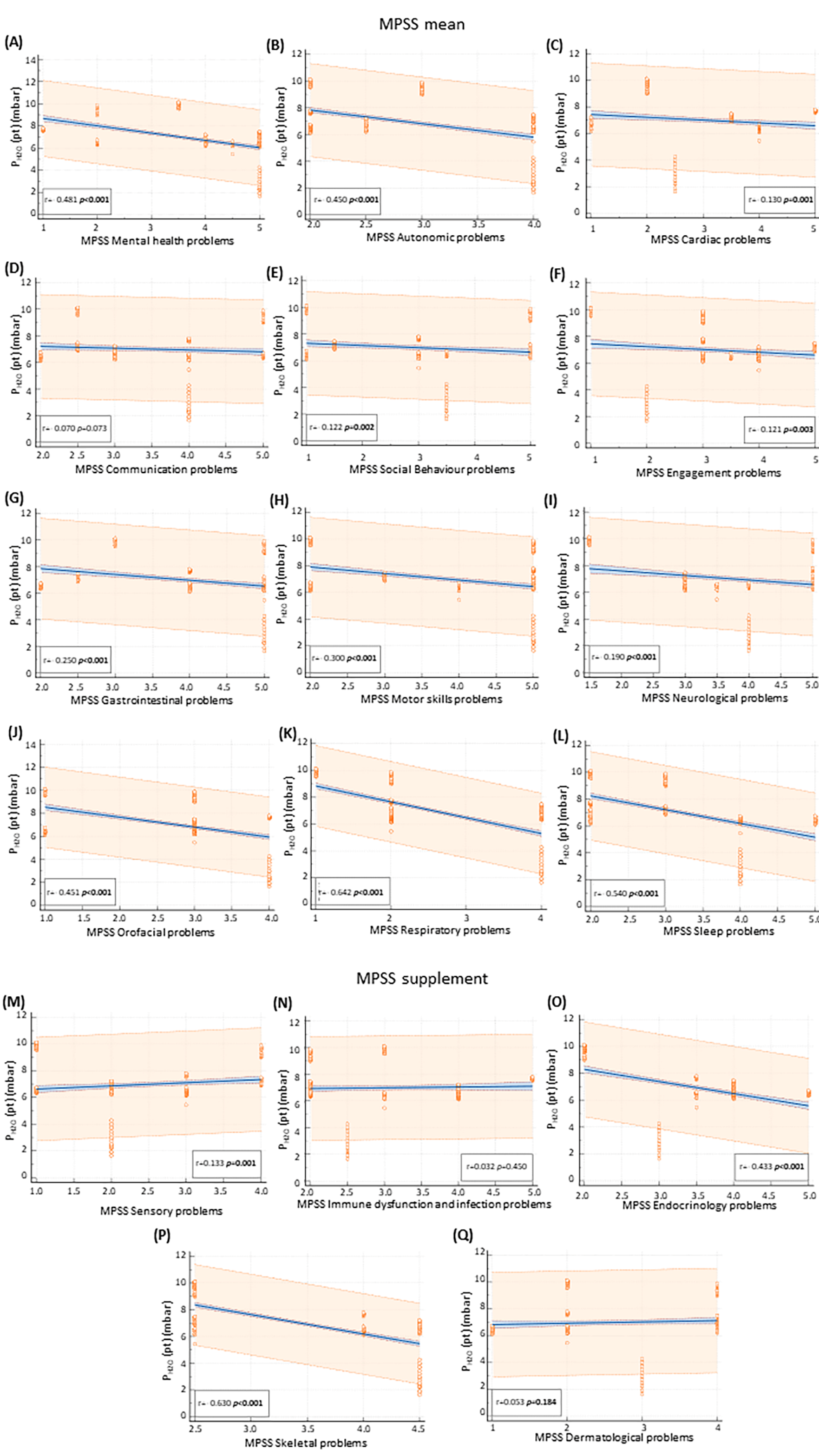


**Supplementary Figure 3.** Correlations between the patient-derived ambient parameter P_H2O_ (pt), and clinical severity (MPSS main and MPSS supplementary scores) in the examined RTT patients (n=10). For the MPSS main scorings, significant inverse correlations with the Mental Health Problems (A), Mental Autonomic problems (B), Orofacial problems (J), Respiratory problems (K), and Sleep problems (L) sub-scores were evidenced (p<0.001). For the MPSS supplementary scorings, statistically significant inverse correlations with the endocrinology problems (O) (p<0.001) and skeletal problems (P) sub-scores were observed (p<0.001).

Abbreviations: P_H2O_ (pt), water vapor partial pressure originating from patients in the bedroom. Bold characters indicate statistically significant differences.
