# Supplementary material for: 24-h continuous non-invasive multiparameter home monitoring of vitals in patients with Rett syndrome by an innovative wearable technology: evidence of an overlooked chronic fatigue status
Source: Front Neurol. 2024 Jun 17;15:1388506. doi: 10.3389/fneur.2024.1388506 (PMC11215834; doi:10.3389/fneur.2024.1388506)
Supplement: Supplementary file 4 [file Data_Sheet_4.DOCX]

Supplementary Material

24-hour continuous noninvasive multiparameter home monitoring of vitals in patients with Rett syndrome by an innovative wearable technology: Evidence of an overlooked chronic fatigue status

**Silvia Leoncini*, Lidia Boasiako, Sofia Di Lucia, Amir Beker, Valeria Scandurra, Aglaia Vignoli, Maria Paola Canevini, Giulia Prato, Lino Nobili, Antonio Gennaro Nicotera, Gabriella Di Rosa, Maria Beatrice Testa Chiarini, Renato Cutrera, Salvatore Grosso, Giacomo Lazzeri, Enrico Tongiorgi, Pasquale Morano, Matteo Botteghi, Alessandro Barducci, Claudio De Felice***

*** Correspondence:** Corresponding Authors: [geniente@gmail.com](mailto:geniente@gmail.com) and [s.leoncini74@gmail.com](mailto:s.leoncini74@gmail.com)


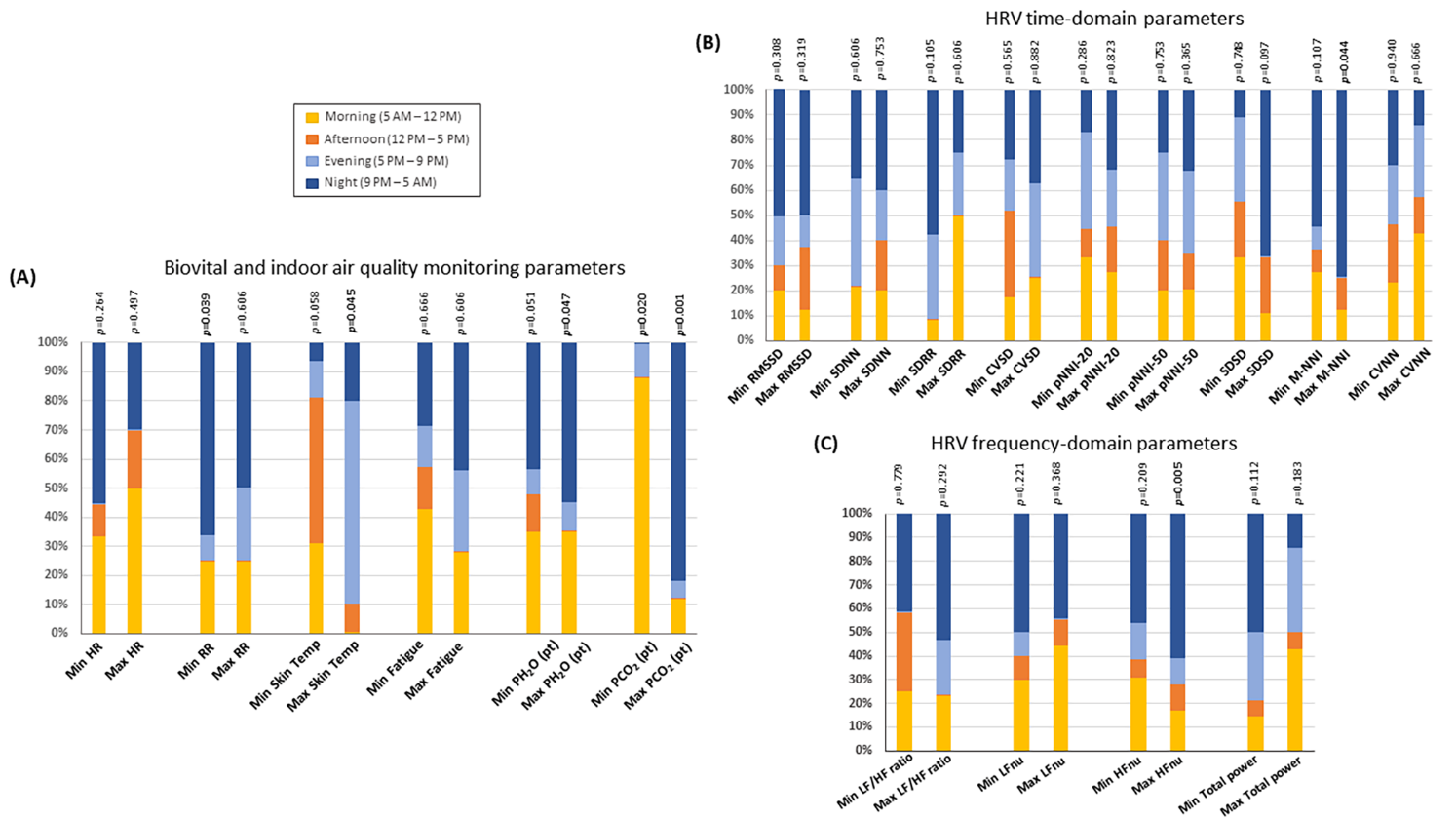


**Supplementary Figure 4.**Twenty-four-hour variation (considered as time distribution of minimum and maximum values) of all the home /IA monitoring variables and two patient-derived parameters P_H2O_ (pt) and P_CO2_ (pt) as indirect markers of sympathovagal imbalance. Statistically significant differences were observed in the time distribution of minimum values for RR (*p*=0.039), P_CO2_ (pt) (*p*=0.020) and maximum values for skin temperature (*p*=0.045), P_H2O_ (pt) (*p*=0.045), and P_CO2_ (pt) (*p*=0.001) (A).No statistically significant difference was evidenced in the HRV time-domain parameters for their daily distributions with the single exception of the maximum value distribution of M-NNI (*p*=0.044) (B).Similar results were found for the HRV frequency-domain parameters with the single exception for the maximum value daily distribution for HFnu (*p*=0.005) (C).

Abbreviations: HR, heart rate; RR, respiratory rate; HRmax %, percentage of maximum heart rate; Skin Temp, skin temperature; RMSSD, Root Mean square of successive RR interval differences; RR intervals, interbeat intervals between all successive heartbeats; SDNN, Standard Deviation of all NN intervals; NN intervals, interbeat intervals with artifacts removed; SDRR, standard deviation of RR intervals; CVSD, RMSSD divided by Mean NNI; pNNI-20, Percentage of  successive RR intervals differing more than 20 ms; pNNI-50, Percentage of  successive RR intervals that differing more than 50 ms; SDSD, SD of successive differences between NN; M-NNI, Mean of NN; CVNNI, SDNN divided by mean NN; LFnu, normalized Low-Frequency power; HFnu, normalized High-Frequency power;  IA, indoor air; PH2O (pt), water vapor partial pressure in the bedroom (patient’s contribution); PCO2 (pt), CO2 partial pressure in the bedroom (patient’s contribution); A.U.; arbitrary units. Note: bold character indicates significant differences.
