# Supplementary material for: 24-h continuous non-invasive multiparameter home monitoring of vitals in patients with Rett syndrome by an innovative wearable technology: evidence of an overlooked chronic fatigue status
Source: Front Neurol. 2024 Jun 17;15:1388506. doi: 10.3389/fneur.2024.1388506 (PMC11215834; doi:10.3389/fneur.2024.1388506)
Supplement: Supplementary file 5 [file Table_1.DOCX]

Supplementary Material

24-hour continuous noninvasive multiparameter home monitoring of vitals in patients with Rett syndrome by an innovative wearable technology: Evidence of an overlooked chronic fatigue status

**Silvia Leoncini*, Lidia Boasiako, Sofia Di Lucia, Amir Beker, Valeria Scandurra, Aglaia Vignoli, Maria Paola Canevini, Giulia Prato, Lino Nobili, Antonio Gennaro Nicotera, Gabriella Di Rosa, Maria Beatrice Testa Chiarini, Renato Cutrera, Salvatore Grosso, Giacomo Lazzeri, Enrico Tongiorgi, Pasquale Morano, Matteo Botteghi, Alessandro Barducci, Claudio De Felice***

*** Correspondence:** Corresponding Authors: [geniente@gmail.com](mailto:geniente@gmail.com) and [s.leoncini74@gmail.com](mailto:s.leoncini74@gmail.com)

**Supplementary Table 1.** Twentyfour-hour continuous home monitoring of RTT patients (*n*=10) as a function beta blocker therapy

| **Variable** | | **Beta-blocker therapy** | | ***p*-value** |
| --- | --- | --- | --- | --- |
|  |  | **No (n=9)** | **Yes (n=1)** |  |
| Wearable monitoring parameters | HR (bpm) | 97.8  [52-153] | 79.7  [46.5-135.5] | 0.1487 |
|  | RR (breaths per min) | 25.9  [12-47.5] | 27.6  [12.0-48.5] | 0.9145 |
|  | HR max % (bpm) | 73.0  [62.2-82.5] | 64.8  [56-73.7] | **<0.0001** |
|  | Skin Temperature (°C) | 38.7  [37.1-39.2] | 35.3  [25.4-37.1] | **<0.0001** |
| HRV time-domain parameters | RMSSD (ms) | 161.7  [14.8-549.5] | 217.8  [46.7-378.5] | 0.2400 |
|  | SDNN (ms) | 133.1  [18.2-432.6] | 149  [37.8-268.9] | 0.5553 |
|  | SDRR (ms) | 19.2  [2.4-52.8] | 14.9  [1.4-49.2] | 0.1264 |
|  | CVSD (ms) | 0.27  [0.03-0.47] | 0.20  [0.04-0.46] | 0.5000 |
|  | pNNI-20 (%) | 63.4  [11.1-93] | 69.9  [34.3-94.1] | 0.4009 |
|  | pNNI-50 (%) | 39.0  [0-88.9] | 46.6  [11.5-82.9] | 0.5877 |
|  | SDSD (ms) | 160.5  [14.5-367.3] | 163.1  [33.8-289.9] | 0.8876 |
|  | M-NNI (ms) | 554.7  [410-977] | 558.5  [435.6-1101.9] | 0.4136 |
|  | CVNNI (ms) | 0.18  [0.03-0.47] | 0.18  [0.03-0.32] | 0.9718 |
| HRV frequency-domain parameters | LFnu (Hz) | 18.3  [17.2-19.2] | 15514.5  [14566.5-16018.0] | **<0.0001** |
|  | HFnu (Hz) | 82.7  [81.6-84.4] | 84.4  [83.3-85.2] | **<0.0001** |
|  | LF / HF ratio | 0.218  [0.195-0.230] | 0.187  [0.177-0.204] | **<0.0001** |
|  | Total power (ms^2^) | 157.8  [32.0-366.0] | 214.3  [105.9-419.8] | **0.0022** |
|  | HR/LF ratio | 5.3  [1.9-7.7] | 0.005  [0.003-0.010] | **<0.0001** |
| Indoor air (IA) monitoring parameters | IA CO_2_ (ppm) | 937  [487.3-1444] | 980.4  [360.5-1689] | 0.4765 |
|  | IA relative humidity (%) | 64.1  [53-73] | 73.7  [61-82] | **<0.0001** |
|  | IA noise level (dB) | 39.0  [33-52.7] | 35.0  [31-50] | 0.1686 |
|  | IA atmospheric pressure (mbar) | 1014.7  [1013.3-1017.9] | 1020.6  [1020.1-1028.3] | **<0.0001** |
|  | IA temperature (°C) | 22.8  [19.3-25.2] | 21.2  [20.1-22.8] | 0.2030 |
|  | P_H2O_ (pt) (mbar) | 6.6  [6.3-9.0] | 7.7  [7.6-7.7] | **<0.0001** |
|  | P_CO2_ (pt) (ppm) | 346.4  [219.2-737.3] | 541.0  [441.8-585.3] | 0.0024 |

Legend: HR, heart rate; RR, respiratory rate; HRmax %, percentage of maximum heart rate; Skin Temp, skin temperature; RMSSD, Root Mean square of successive RR interval differences; RR intervals, interbeat intervals between all successive heartbeats; SDNN, Standard Deviation of all NN intervals; NN intervals, interbeat intervals from which artifacts have been removed; SDRR, standard deviation of RR intervals; CVSD, RMSSD divided MeanNNI; pNNI-20, Percentage of successive R-R intervals that differ by more than 20 ms; pNNI-50, Percentage of successive R-R intervals that differ by more than 50 ms; SDSD, SD of successive differences between NN; M-NNI, Mean of NN; CVNNI, SDNN divided by mean NN; LFnu, normalized Low-Frequency power; HFnu, normalized High-Frequency power; IA, indoor air; P_H2O_ (pt), water vapor partial pressure originating from patients in the bedroom; P_CO2_ (pt), CO_2_ partial pressure originating from patients in the bedroom; A.U., arbitrary units.
